# Radiation adaptive response for constant dose-rate irradiation in high background radiation areas

**DOI:** 10.1007/s00411-024-01093-0

**Published:** 2024-10-29

**Authors:** Ernest Bugała, Krzysztof Wojciech Fornalski

**Affiliations:** Faculty of Physics, Warsaw University of Technology, ul. Koszykowa 75, Warszawa, 00-662 Poland

**Keywords:** Adaptive response, Radioadaptation, High background radiation areas, HBRA, Chromosomal aberrations, Cancer mortality, Cancer incidence, Low dose radiation

## Abstract

The presented paper describes the problem of human health in regions with high level of natural ionizing radiation in various places in the world. The radiation adaptive response biophysical model was presented and calibrated for the special case of constant dose-rate irradiation. The calibration was performed for the data of residents of several high background radiation areas, like Ramsar in Iran, Kerala in India or Yangjiang in China. Studied end-points were: chromosomal aberrations, cancer incidence and cancer mortality. For the case of aberrations, among collected publications about 45% have shown the existence of adaptive response. Average reduction of chromosomal aberrations was ∼ 10%, while for the case of cancer incidence it was ∼ 15% and ∼ 17% for cancer mortality (each taking into account only results showing adaptive response). Results of the other 55% of data regarding chromosomal aberrations have been tested with the LNT (linear no-threshold) hypothesis, but results were inconsistent with the linear model. The conditions for adaptive response occurrence are still unknown, but it is postulated to correlate with the distribution of individual radiosensitivity among members of surveyed populations.

**Supplementary Information:**

The online version contains supplementary material available at 10.1007/s00411-024-01093-0.

## Introduction

Ionizing radiation is a phenomenon that each of us encounters every day. It is omnipresent on Earth and thus, there is no single place where the dose-rate is 0 Gy per unit time. Moreover, there are areas around the globe where the level of natural background radiation is several times higher than the world average. These areas are called high background radiation areas, abbreviated as HBRA, and include Ramsar in Iran, Kerala in India and Yangjiang in China. It is very difficult to unequivocally determine the general and universal dose-effect relationship among the inhabitants of aforementioned HBRAs using epidemiological methods (Hendry et al. 2009; Lubin and Boice 1997; Darby et al. 2005; Dobrzyński et al. 2015, 2018; Kendall et al. 2013; Spycher et al. 2015; Mazzei-Abba et al. 2021; David et al. 2021, 2022). One of the possible explanations is the frequent lack of statistical power of the studies, however, many researchers state that people constantly exposed to elevated (relatively to world average) radiation doses can manifest adaptive response (Guéguen et al. 2019; Tapio and Jacob 2007; Dimova et al. 2008). This effect is based on stimulating the body’s natural protective mechanisms by low doses (and low dose-rates) of ionizing radiation. Its mechanisms involve (among others) regulation of the cell cycle, induction of the apoptosis (programmed cell death), elevated concentration of enzymes responsible for DNA repair or removal of reactive oxygen species, and reduction of the number of mutations (Guéguen et al. 2019).

It should be noted that the occurrence (or lack) of the adaptive response and the degree of its manifestation depends on several yet unidentified individual factors. This means, that the adaptive response may manifest itself stronger in some individuals than others, and in some cases it may not appear at all (Tapio and Jacob 2007). Generally, radiation effects on the human organism are highly multifactorial phenomena and the different molecular mechanisms involved in each individual are therefore very difficult to grasp by modelling on a global population scale. However, such models could be potentially helpful from the point of view of radiation protection. Therefore, the following work aims to calibrate a dedicated model describing the probability of adaptive response occurrence among HBRA inhabitants and to verify the calculated parameters. The crucial aim of this paper is not to prove the universality of radiation adaptive response effect in humans but to assess the probability function of its occurrence in relation to radiation dose (or dose-rate) in the case when it is present.

As already mentioned HBRAs are regions where the levels of background radiation are statistically significant larger than the average of other non-HBRAs. There are several definitions of HBRAs, reviewed by (Hendry et al. 2009). The elevated levels of radiation are the result of natural conditions, e.g. the presence of radon or naturally occurring radioactive material, and are not caused by human activity. Because of their origin, these areas are sometimes called HNBRA, where the ‘N’ stands for ‘natural’.

The world average annual effective dose originating from background radiation is 2.4 mSv/year (UNSCEAR 2000). The natural background radiation levels vary depending on location, usually in the range of 1 to 13 mSv/year (UNSCEAR 2008). This may be due to geological properties (presence of uranium- or thorium-rich rocks), geographical (dependence of cosmic radiation levels on latitude) aspects or simply by altitude. In the HBRAs, annual doses may be as high as 260 mSv, but average doses are usually defined as two times higher than the world average (Hendry et al. 2009). Additionally, one has to be aware of differences in radiation qualities in the different HBRAs. Different radiation qualities have different biological effects which can bias final conclusions of observational studies. Most researches are dealing with mixed irradiation, from radon progeny and external gamma radiation, however, every pooled or meta-analysis needs to use some common quantity, like personal effective dose.

According to the LNT (linear no-threshold) model, which is used for predicting effects at low doses and low dose-rates, the risk of stochastic effects increases linearly with the received dose of ionizing radiation (Laurier et al. 2023; Wojcik and Zölzer 2024). This approach was implemented as a basis of international radiation protection standards. It means that any dose, even the smallest one, may increase the risk of cancer such as leukaemia. Following that postulate, any inhabitant of the HBRAs is more at potential risk of cancer than a person living in an area of average background radiation. Thus, we should expect an increased incidence in solid cancer and leukaemia among the HBRA inhabitants, which is not the case. Several studies show that the frequency of cancer incidence and mortality in the high background radiation areas in relation to the areas of normal background radiation levels are actually the same or smaller (but usually statistically insignificant) (Dobrzyński et al. 2015; David et al. 2021, 2022; Tao et al. 2000; Nair et al. 2009; HBRRG 1980; Cheryian et al. 1999; Hayata et al. 2000; Mortazavi et al. 2003a; Ramachandran et al. 2013; Syaifudin et al. 2018). One of the possible explanations of this phenomenon is the radiation adaptive response.

## Radiation adaptive response

Adaptive response effect describes adaptation of an organism to often detrimental environmental conditions, by induction or enhancement of the organism’s defence mechanisms. Many studies show that these may include improved detoxification of free radicals, enhancement of DNA repair, immune responses and regulation of the cell cycle (Guéguen et al. 2019; Tapio and Jacob 2007; Dimova et al. 2008; UNSCEAR 1994, 2021). Especially the first explanation seems to be of particular relevance as radiation adaptive responses are believed to involve the management of radiation induced oxidative stress by stimulation of antioxidants (Paraswani et al. 2018; Matsumoto et al. 2007; Lall et al. 2014), ATM nucleoshuttling (Devic et al. 2020), as well as mitochondrial and immunological responses (Paraswani et al. 2018; Matsumoto et al. 2007; Lall et al. 2014; Toledo et al. 2006; Bravard et al. 1999; Averbeck 2023; Hussien 2023). A particular example of radiation adaptive response is the Raper-Yonezawa effect (also called priming dose effect) (Fornalski et al. 2022a). It consists of gaining a short term (lasting few hours, days or weeks) elevated resistance to radiation after receiving a small priming dose prior to a larger challenging dose. Thanks to this, despite the total dose being larger (priming + challenging dose), the effects on the organism are less detrimental in comparison to receiving only the challenging dose. This provokes the question what would happen if the priming dose was not delivered as a short-term dose pulse, but in the form of constantly elevated background dose. Studies carried out in HBRAs aim to answer this question. Indeed, some of the studies show that the radiation adaptive response is present in a HBRA (HBRRG 1980; Cheryian et al. 1999; Hayata et al. 2000; Mortazavi et al. 2003a; Ramachandran et al. 2013; Syaifudin et al. 2018). It manifests itself as a reduced frequency of chromosomal aberrations in cells of HBRA inhabitants and as a reduced cancer incidence and mortality. The areas taken into account in each study are similar geographically and culturally, and often cohorts and areas of the same country or the same region serve as a control group or control area (CA). Such studies have not always demonstrated radioadaptive responses (Ghiassi-Nejad et al. 2004; Chen and Wei 1991; Jiang et al. 2000; Zhang et al. 2003, 2004; Wang et al. 1990; Zakeri et al. 2011). In the present work, the percentage of studies showing radioadaptive responses has been estimated as being 45%. The other 55% of the studies did not detect the presence of radiation adaptive response, which seems to be in line with the concept of the LNT model. Considering that the effects of ionizing radiation on an organism depend on individual factors (every person exhibits a different radioresistance and is more or less prone to the effects of ionizing radiation), the radioadaptive response will not occur in every person and under all conditions, or may vary in intensity.

In the following, we present a theoretical approach to describe the radiation adaptive response (Fornalski et al. 2024). The radioadaptive response is discussed with regard to chromosomal aberrations induced in relation to cancer incidence and mortality.

## Methods – model description

The presented model describes the probability function of the radiation adaptive response (*p*_*AR*_) in relation to radiation dose (*D*) which was delivered some time (*t*) ago:

1 $$\:{p}_{AR}={\alpha\:}_{0}{D}^{2}{t}^{2}\text{exp}\left(-{\alpha\:}_{1}D-{\alpha\:}_{2}t\right)$$

where *α*_*0*_, *α*_*1 *_and *α*_*2*_ are calibration parameters. Details of the calculations were described previously (Fornalski et al. 2022a, 2024; Dobrzyński et al. 2019).

**Fig. 1:**
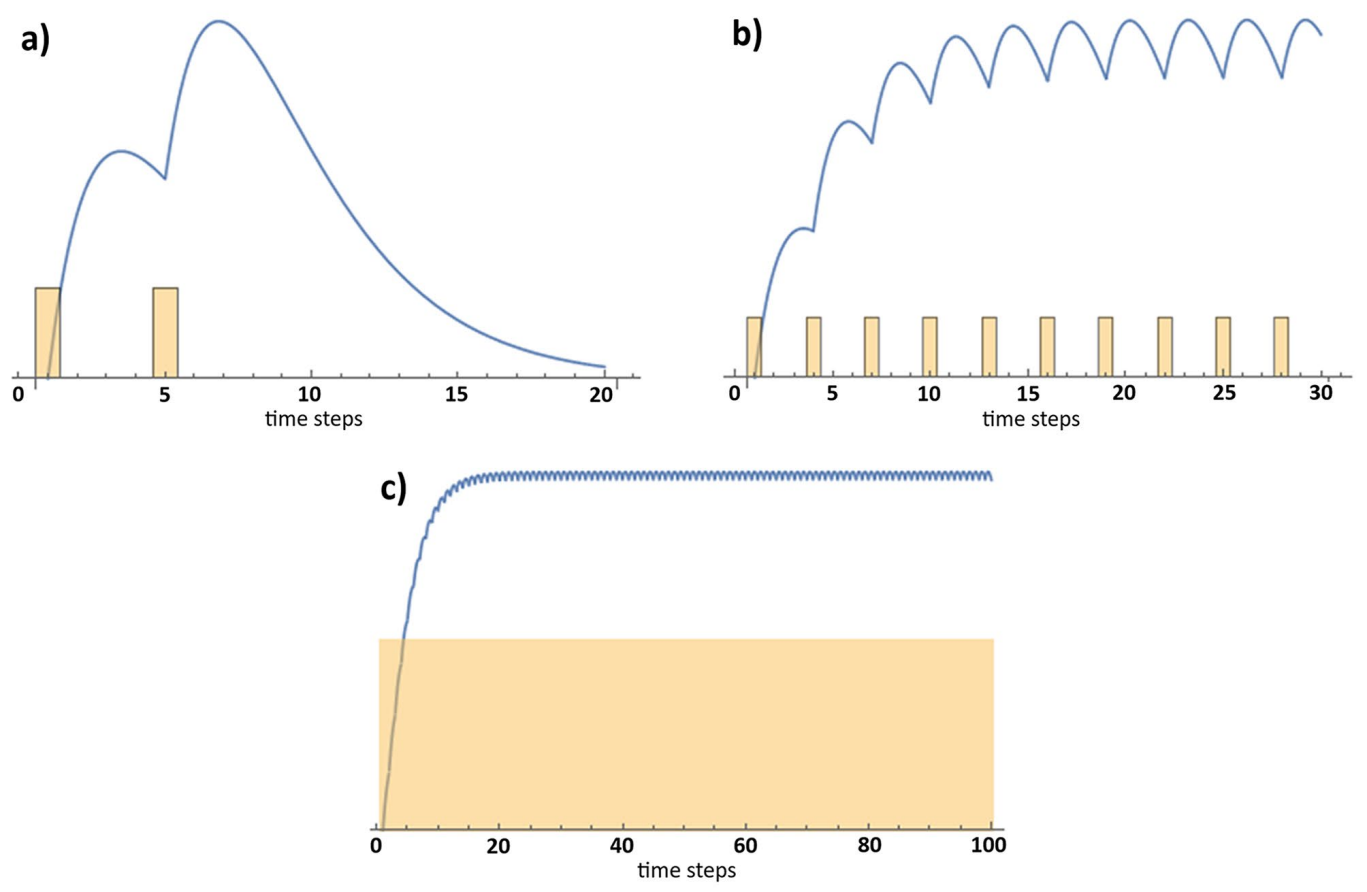
Schematic presentation of the adaptive response probability for (**a**) two low dose pulses, (**b**) multiple low dose pulses (so called “radiation training”), and (**c**) constant dose-rate. Blue line represents the joint probability function, while the yellow bars represent dose pulses or background dose-rate. Horizontal axis represents time steps

The model is universal in its application, because it can simulate the adaptive response originating from one or several dose pulses (Fornalski et al. 2022a, b). Simulated probability distribution are schematically depicted in Fig. 1 for a few exemplary scenarios. It should be noted that the graphs present only qualitative examples of the model. In the case of chronic irradiation with constant or varying dose-rate, the probability function of the radiation adaptive response will saturate after some time (Fornalski 2022), see Fig. 1c.

The exemplary case presented in Fig. 1a is a sum of two functions given by Eq. (1) for both dose pulses. One can observe that the second dose significantly improves the joint probability function of the adaptive response appearance. Analogically, Fig. 1b represents multiple doses as a sum of several *p*_*AR*_ separated by some small time intervals, *Δt*. The joint probability function (in the moment of *t*) is a sum of all single functions given by Eq. (1) generated by the dose pulses (*D = const*) received at *Δt*, *2·Δt*, *3·Δt*, *…*, and *t* time ago:

2 $$\begin{aligned}{P_{AR}}\left( t \right) &  = {\alpha _0}{D^2}{e^{ - {\alpha _1}D}}\sum \limits_{\varDelta t = 0}^{t} {\left( {t - \varDelta t} \right)^2}{e^{ - {\alpha _2}\left( {t - \varDelta t} \right)}} \\ &  = \bar c\sum \limits_{\varDelta t = 0}^{t} \varDelta {t^2}{e^{ - {\alpha _2}\varDelta t}} \\ \end{aligned} $$

which was applied also in Fig. 1c, where *Δt* is very small. In the situation when *Δt* → 0, one gets the continuous form of Eq. (2) as:

3 $${P_{AR}}(t) = \int_0^t {{p_{AR}}\,dt = {\xi _D}\left( {1 - {e^{ - {\alpha _2}t}}\left[ {{1 \over 2}{{({\alpha _2}\,t)}^2} + {\alpha _2}\,t + 1} \right]} \right)} $$

where $\:{\xi\:}_{D}=2\:\overline{c}\:{\alpha\:}_{2}^{-3}$ and all calculations are presented in (Fornalski et al. 2022a). Please note that in order to apply the model to a situation of chronic irradiation with constant dose-rate, one would need additionally to change dose $\:D$ to dose-rate $\:\dot{D}$ (which changes all units as well). Moreover, while modelling the adaptive response among the HBRA inhabitants, to further simplify the equation, one should assume very long time of irradiation where Eq. (3) saturates to some time-constant value (see Fig. 1c). Such assumption is correct, because many of the HBRA inhabitants live in those areas for decades or even several generations (HBRRG 1980); it is achieved mathematically by going with time to infinity (Fornalski 2022). Then the equation describing the time-constant value of saturated long-time adaptive response is as follows:

4 $$\:{P}_{C}=\underset{T\to\:\infty\:}{\text{lim}}{\int\:}_{0}^{T}{p}_{AR}\left(\dot{D},t\right)dt=2\beta\:\frac{{\alpha\:}_{0}^{{\prime\:}}}{{\alpha\:}_{2}^{3}}{\dot{D}}^{2}\text{e}\text{x}\text{p}(-{\alpha\:}_{1}^{{\prime\:}}\dot{D})\:\:$$

where $\:\beta\:$ is a normalizing constant applied, so that the maxima of the functions 4 and 1 (or 3) are equal. Moreover, it should be noted that parameters $\:{\alpha\:}_{0}$ and $\:{\alpha\:}_{1}$ had been substituted with $\:{\alpha\:}_{0}^{{\prime\:}}$ and $\:{\alpha\:}_{1}^{{\prime\:}}$. This is caused by changing the dose $\:D$ into dose rate $\:\dot{D}$ (change of units). New constants are thus: $\:{\alpha\:}_{0}^{{\prime\:}}={\alpha\:}_{0}{\theta\:}^{2}$ and $\:{\alpha\:}_{1}^{{\prime\:}}={\alpha\:}_{1}\theta\:$, where $\:\theta\:$ denotes saturation time of the radiation adaptive response probability. Again, in order to simplify the notation, one can define new constant $\:{\alpha\:}_{3}=2\beta\:\frac{{\alpha\:}_{0}^{{\prime\:}}}{{\alpha\:}_{2}^{3}}.$ Then Eq. 4 becomes (Fornalski et al. 2024):

5 $$\:{P}_{C}={\alpha\:}_{3}{\dot{D}}^{2}\text{exp}(-{\alpha\:}_{1}^{{\prime\:}}\dot{D})$$

which is a function of dose-rate ($\:\dot{D}$) only, but not a function of the time of irradiation (*t*). After the detailed description presented above the question arises how this model can be applied to experimental data. The answer is presented in publication (Fornalski 2022) as an equation for reduction factor (called also a repair effectiveness), $\:R$. This allows for including both reduction of chromosome damage and reduction of cancer incidence or mortality. Reduction factor *R* denotes the percentage of how much the adverse effects of ionizing radiation have been reduced. $\:R$ is calculated as:

6 $$\:R=1-\text{e}\text{x}\text{p}(-{P}_{C})$$

Second equation used for experimental calculation of reduction factor in HBRAs is:

7 $$\:R=1-\frac{{I}_{HBRA}}{{I}_{CA}}$$

where $\:{I}_{HBRA}$ and $\:{I}_{CA}$ denote the incidence rate of modelled end-points (chromosomal aberrations, cancer incidence or mortality) in a high background radiation area and a control area, respectively. The above relationship provides a link between the theoretical model describing the probability of radioadaptive response and real experimental data obtained.

One important drawback of the model should be noted here. It only allows for modelling of the radioadaptive response and does not take into account any cases where such response was absent, meaning there is an increase in observed end-points with dose. Because of the properties of $\:\text{exp}\left(-x\right)$ and the fact that $\:\dot{D}>0$, values of reduction factor $\:R$ will always be positive, thus datasets where $\:{I}_{HBRA}>{I}_{CA}$ should not be taken into account. This means that the phenomenon of radiation adaptive response may be modelled only if it can be observed, e.g. such data exist. This leads to scientific bias, because it automatically eliminates studies that did not demonstrate radioadaptive response. In order to eliminate that bias, not only publications indicating the existence of radioadaptive response, but also publications that disproved its existence were collected. Then, it was calculated how many percent of the publications indicated the existence of radioadaptive response. It should be noted, that the appearance of the radioadaptive response had been noted in 45% of the gathered publications. The review paper (Tapio and Jacob 2007) states that the radiation adaptive response occurs among 50–78% of the population, but the studies analysed there were carried out in vitro with the Raper-Yonezawa irradiation scheme, thus it presents a different type of adaptive response assessment than the one discussed here. An overview of publications included in the statistical analysis above is presented in detail in the Supplementary Material, as well as in Table 1.

**Table 1:** Summary of studies regarding chromosomal aberrations among the HBRA inhabitants that observed or disproved the radiation adaptive response (see supplementary material)

Publication	Occurrence of the radiation adaptive response
Cheryian et al. 1999	4 results for, 1 against
Hayata et al. 2000	Yes
Mortazavi et al. 2003a	Yes
Ramachandran et al. 2013	Yes
High Background Radiation Research Group, China, 1980 (HBRRG 1980)	Yes
Syaifudin et al. 2018	Yes
Ghiassi-Nejad et al. 2004	No
Chen and Wei 1991	No
Jiang et al. 2000	No
Zhang et al. 2004	No
Wang et al. 1990	No
Zakeri et al. 2011	No
Zhang et al. 2003	No

## Materials

Prior to describing the publications and the method for calibration of the model, the following data are needed: First, to calibrate the model, values of annual doses from background radiation received by the HBRA and the CA inhabitants. Second, data concerning the frequency (or incidence) of chromosomal aberrations (or other end-points described further). Each data should be accompanied by relevant details on uncertainty. One of the main tasks in this work was to unify the data in a way that could be applied to the model.

### Chromosomal aberrations – detrimental effects

As mentioned earlier, in order to compensate for the model’s scientific bias, i.e. the ability to include only datasets where the radioadaptive response is observable, data disproving the adaptive response have been presented in Table 2 and in detail in the Supplementary Material.

**Table 2:** Data gathered from publications disproving radiation adaptive response, see supplementary material. Description of symbols: $\:\dot{D}$ is a dose-rate, $\:{I}_{HBRA}$ is incidence of chromosomal aberrations in HBRAs, $\:{I}_{CA}$ is incidence of chromosomal aberrations in control areas, and RR corresponds to the relative risk. All uncertainties represent one standard deviation

Publication	$\:{\dot{D}}_{HBRA}$ (mSv/year)	$\:{\dot{D}}_{CA}$ (mSv/year)	$\:{\Delta\:}\dot{D}$ (mSv/year)	$\:{I}_{HBRA}$	$\:{I}_{CA}$	$\:RR$
Ghiassi-Nejad et al. 2004	13 ± 12	2.30 ± 0.09	10 ± 12	0.0599 ± 0.0035	0.0150 ± 0.0022	3.98 ± 0.64
Chen and Wei 1991	2.10 ± 0.53	0.75 ± 0.19	1.35 ± 0.56	0.19 ± 0.10	0.05 ± 0.05	4.9 ± 5.4
	2.70 ± 0.68		0.33 ± 0.12	10 ± 22		1.95 ± 0.72
Jiang et al. 2000	3.70 ± 0.44	0.704 ± 0.075	2.99 ± 0.44	6.3 ± 4.5	3.4 ± 2.1	1.9 ± 1.8
Zhang et al. 2004	2.71 ± 0.31	0.72 ± 0.12	1.99 ± 0.33	12.4 ± 5.2	10.0 ± 3.8	1.24 ± 0.70
Wang et al. 1990	2.89 ± 0.72	1.00 ± 0.25	1.9 ± 1.0	0.44 ± 0.07	0.23 ± 0.05	1.90 ± 0.52
Zakeri et al. 2011	4.4 ± 2.8	1.27 ± 0.26	3.1 ± 2.0	0.86 ± 0.44	0.23 ± 0.17	3.7 ± 3.4
Zhang et al. 2003	2.75 ± 0.33	0.683 ± 0.093	2.07 ± 0.34	0.0035 ± 0.0019	0.0030 ± 0.0019	1.18 ± 0.99
Cheryian et al. 1999	19.1 ± 4.8	1.15 ± 0.29	17.9 ± 4.8	9.0 ± 1.3	8.01 ± 0.71	1.12 ± 0.19

### Chromosomal aberrations – radioadaptation effects

Data used for calibration originate from six publications that showed radioadaptive response (see Table 3) (HBRRG 1980; Cheryian et al. 1999; Hayata et al. 2000; Mortazavi et al. 2003a; Ramachandran et al. 2013; Syaifudin et al. 2018). The first two of them describe studies conducted in Yangjiang - a region of China known for its high natural background radiation areas. Studies shown in the next two publications concern newborns from the Kerala state in India, whose parents lived in the local HBRAs. The fifth publication from the list concerns one of the most widely known places in Iran – Ramsar, famous for the highest background radiation doses in the world (Laurier et al. 2023). The publications are described in detail in Supplementary Material.

**Table 3:** Data used for model calibration in relation to chromosomal aberrations (see for further details supplementary material). Description of symbols: $\:\dot{D}$ is a dose-rate, $\:{I}_{HBRA}$ is incidence of chromosomal aberrations in HBRAs, $\:{I}_{CA}$ is incidence of chromosomal aberrations in control areas, and *RR* corresponds to the relative risk. All uncertainties represent one standard deviation

Publication	$\:{\dot{D}}_{HBRA}$ (mSv/year)	$\:{\dot{D}}_{CA}$ (mSv/year)	$\:{\Delta\:}\dot{D}$ (mSv/year)	$\:{I}_{HBRA}$ (per 100 cells)	$\:{I}_{CA}$ (per 100 cells)	$\:RR$
HBRRG 1980	2.31 ± 0.58	0.96 ± 0.23	1.39 ± 0.62	0.270 ± 0.036	0.274 ± 0.036	0.98 ± 0.18
Hayata et al. 2000	3.01 ± 0.29	0.621 ± 0.020	2.39 ± 0.29	1.32 ± 0.18	1.44 ± 0.26	0.92 ± 0.21
Cheryian et al. 1999	1.87 ± 0.47	1.15 ± 0.29	0.72 ± 0.55	0.0750 ± 0.0038	0.0801 ± 0.0071	0.936 ± 0.096
	4.7 ± 1.0		2.9 ± 1.1	0.0787 ± 0.0066		0.98 ± 0.12
	7.5 ± 1.9		6.3 ± 1.9	0.064 ± 0.014		0.80 ± 0.18
	31.4 ± 7.9		30.3 ± 7.9	0.064 ± 0.018		0.80 ± 0.23
Ramachandran et al. 2013	1.81 ± 0.45	1.19 ± 0.30	0.62 ± 0.54	0.0825 ± 0.0037	0.0929 ± 0.0055	0.888 ± 0.066
	4.1 ± 1.0		2.9 ± 1.1	0.0870 ± 0.0061		0.936 ± 0.086
	18.0 ± 4.5		16.8 ± 4.5	0.0794 ± 0.0076		0.855 ± 0.096
Mortazavi et al. 2003a	53 ± 21	0.447 ± 0.080	52 ± 21	1.29 ± 0.36	1.60 ± 2.4	0.80 ± 0.26
Syaifudin et al. 2018	5.0 ± 1.2	1.42 ± 0.36	3.6 ± 2.5	0.082 ± 0.024	0.125 ± 0.056	0.65 ± 0.35

The incidences (*I*_*HBRA*_ and *I*_*CA*_) of chromosomal aberrations given in Table 3 are presented as aberrations per 100 cells, while in the original papers they were presented as aberrations per *n* number of cells (this is unified here). Differences in aberration incidence at similar dose values can be noted. They may be due to differences in methods of counting aberrations or growing cells in cultures. One should note that the oldest and most recent papers were published over 30 years apart. Since 1980 the methods of conducting such studies may have changed, and their accuracy has improved through the years. Still, in order to calibrate the model only the ratio of incidence is used. Thus, if both incidence groups were studied using the same method (which is the case), then using the ratio mitigates methodologic differences.

One has to note that more studies on aberrations in HBRAs were published in scientific literature, for example (Su et al. 2018; Jain et al. 2017; Ramachandran et al. 2017; Mohammadi et al. 2006; Talebian et al. 2020; Shimura and Kojima 2018; Mortazavi et al. 2019). However, to provide comparable data within the presented paper, it was decided to limit the method of studies included in the dataset to those that assessed the total number or incidence of chromosomal aberrations (Tables 2 and 3). Rest of studies, which can be found in the literature, use different methods of searching for adaptive response (i.e. PCR, antioxidative capacity, micronucleus or gamma-H2AX assay), thus for the sake of clarity of the compared data, they were not included in this analysis.

### Cancer morbidity and mortality effects

Aside from data concerning chromosomal aberrations, also other end-points were studied. An attempt was made to calibrate the model to describe (separately) a reduction of cancer incidence and cancer mortality (see Table 4). Similarly to publications on chromosomal aberrations, publications describing studies comparing cancer incidence or mortality in HBRA and CA were analysed (see details in Supplementary Material). Then, the additional dose $\:{\Delta\:}\dot{D}$ was calculated as well as relative risk $\:\left(RR\right)$ along with respective uncertainties. Only solid cancer data were considered due to the different mechanism of formation, development and treatment than leukaemia.

**Table 4:** Data used for model calibration in relation to cancer incidence and cancer mortality, see supplementary material. All uncertainties represent one standard deviation

Publication	Type of endpoint	$\:{\dot{D}}_{HBRA}$ (mGy/year)	$\:{\dot{D}}_{CA}$ (mGy/year)	$\:{\Delta\:}\dot{D}$ (mGy/year)	$\:RR$
Nair et al. 1999	Cancer incidence	4.6 ± 1.6	0.99 ± 0.35	3.6 ± 1.7	1.11 ± 0.27*
		3.4 ± 1.2		2.4 ± 1.3	0.82 ± 0.28
		2.75 ± 0.99		1.8 ± 1.0	0.73 ± 0.19
		3.8 ± 1.4		2.8 ± 1.4	0.81 ± 0.24
		1.15 ± 0.41		0.15 ± 0.54	0.88 ± 0.23
		1.72 ± 0.61		0.73 ± 0.70	0.84 ± 0.28
		2.26 ± 0.81		1.26 ± 0.88	0.82 ± 0.21
		1.26 ± 0.45		0.27 ± 0.57	0.79 ± 0.21
		1.95 ± 0.69		0.96 ± 0.78	0.78 ± 0.24
		1.49 ± 0.53		0.50 ± 0.64	0.83 ± 0.22
		2.52 ± 0.91		1.53 ± 0.98	0.93 ± 0.23
Nair et al. 2009		1.5 ± 0.2	0.8 ± 0.1	0.7 ± 0.5	0.92 ± 0.08
		3.2 ± 0.6		2.4 ± 0.8	0.89 ± 0.08
		6.8 ± 1.0		6.0 ± 1.0	0.88 ± 0.10
		14.4 ± 4.2		13.6 ± 2.1	0.91 ± 0.13
Nair et al. 1999	Cancer mortality	4.6 ± 1.6	0.99 ± 0.35	3.6 ± 1.7	0.90 ± 0.36
		3.4 ± 1.2		2.4 ± 1.3	0.48 ± 0.30
		2.75 ± 0.99		1.8 ± 1.0	0.77 ± 0.30
		3.8 ± 1.4		2.8 ± 1.4	0.99 ± 0.43
		1.15 ± 0.41		0.15 ± 0.54	0.76 ± 0.31
		1.72 ± 0.61		0.73 ± 0.70	1.04 ± 0.50*
		2.26 ± 0.81		1.26 ± 0.88	0.70 ± 0.29
		1.26 ± 0.45		0.27 ± 0.57	0.74 ± 0.31
		1.95 ± 0.69		0.96 ± 0.78	0.83 ± 0.39
		1.49 ± 0.53		0.50 ± 0.64	1.01 ± 0.40*
		2.52 ± 0.91		1.53 ± 0.98	0.95 ± 0.37
Wei et al. 1990		5.4 ± 1.3	2.01 ± 0.50	3.36 ± 1.43	0.93 ± 0.06
					0.96 ± 0.08
Tao et al. 2000		6.4 ± 1.6	2.4 ± 0.6	4.0 ± 1.7	0.96 ± 0.09

* - data excluded from set on the basis of $\:RR>1$

## Results

### Detrimental effects

**Fig. 2:**
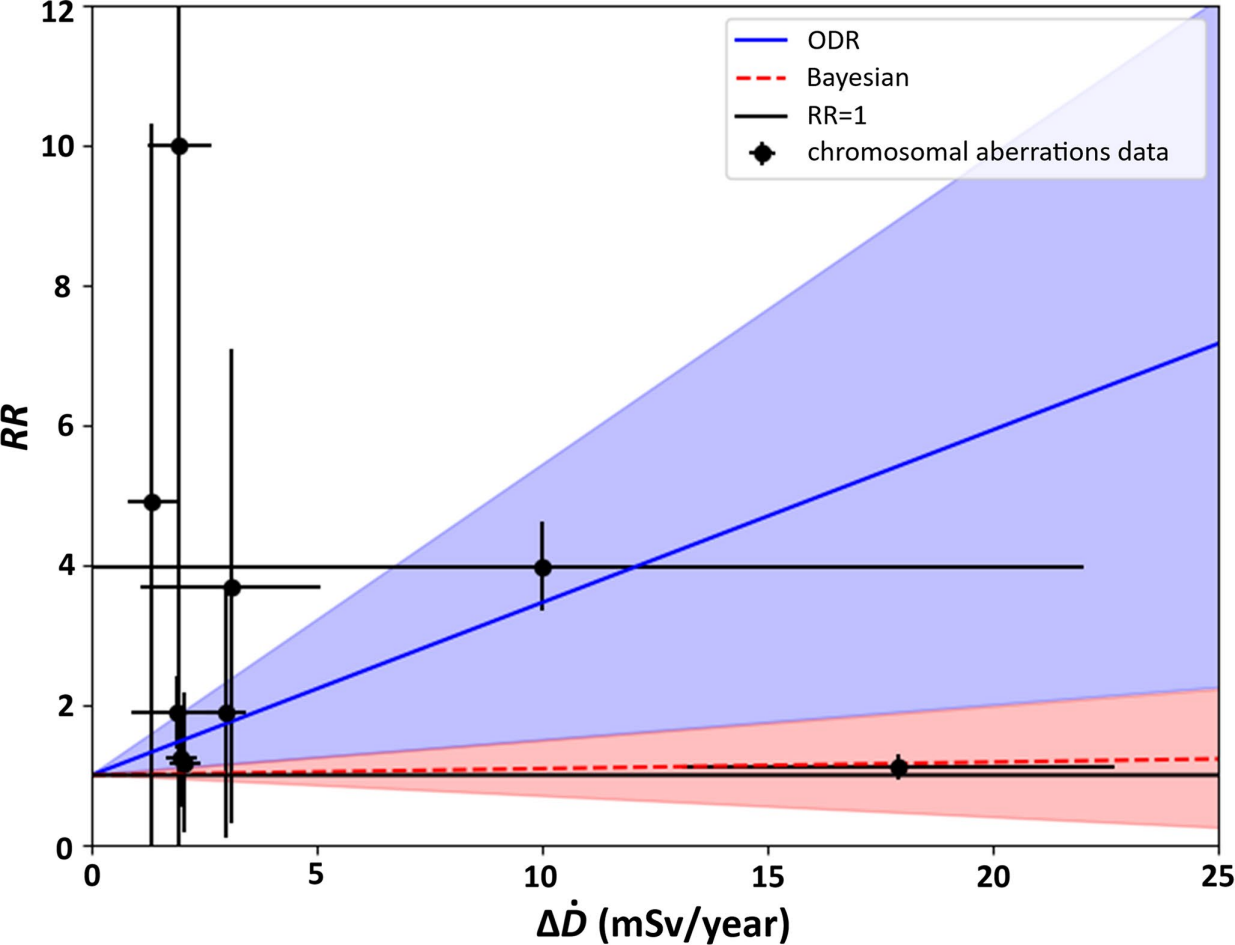
Plot of data regarding relative risk (RR) for chromosomal aberrations (black points with uncertainties) originating from studies disproving the existence of radioadaptive response (e.g. the data which show detrimental effects only – Table 2) with LNT fit for two different fitting algorithms: classical best fit by ODR method (upper solid blue line) and robust Bayesian one (lower dashed red line). Each line is surrounded by its uncertainty area. All uncertainties represent one standard deviations

Data presented in Table 2 were plotted and presented in Fig. 2. Because of radiation protection standards, those data were tested according to the relevance with the LNT model and a linear function $\:RR=a\:{\Delta}\dot{D}+1$ was fitted to the data. The intercept was set as 1, because it was assumed that for excess dose $\:{\Delta}\dot{D}=0$ mSv/year (thus equal to normal background radiation dose), relative risk should be 1 (if there is no excess dose, then the additional risk should be zero). Obtained values of the slope are: for the ODR (Orthogonal Distance Regression) method: $\:a=0.25\pm\:0.20$ year/mSv and for the Bayesian method: $\:a=0.01\pm\:0.04\:$ year/mSv (see Table 5). The fitted linear functions are indicated in Fig. 2. Blue region represents the uncertainty of the ODR fit method, whereas the red region represents the uncertainty of the Bayesian fit (one standard deviation).

The blue line was fitted using Python script applying the ODR fitting algorithm, taking into account the uncertainties of both excess dose and relative risk values. Aside from the fitted line, also the calculated (by the script) regions of uncertainty have been marked. The red line along with the red region refers to the Bayesian fit and its uncertainty. The slope of that function was calculated with ROOT script implementing the robust Bayesian regression analysis (Fornalski 2015). This method was selected, because it has previously been used for the verification of the dose-effect relationship models (Dobrzyński et al. 2015, 2018; Fornalski 2015).

Exact value of the slope calculated by the Bayesian fit is $\:a=0.0093\:\pm\:\:0.0396$ (one standard deviation). This means that the uncertainty is four times larger than the value. and the presented risk increase is statistically insignificant. Thus, the application of the LNT model is inconsistent regarding the calculated slope, the different results obtained by the two independent statistical methods, and the large scatter of data points in Fig. 2.

**Table 5:** Slope values fitted with two best-fit methods, see Fig. 2. All uncertainties represent one standard deviation

Fitting method	$\:a$ (year/mSv)
ODR (Orthogonal Distance Regression)	0.25 ± 0.20
Robust Bayesian regression	0.01 ± 0.04

### Radioadaptation effects

The model (thoroughly described in Sect. 3) was calibrated with the use of dedicated Python numerical script. For the sake of transparency and reproducibility of the results, data used for calibration had the same precision as presented in Table 3. The same approach was assumed for the data regarding cancer incidence and mortality (Table 4).

After substituting Eq. 5 into 6 and comparing the right-hand sides of the Eqs. 6 and 7, Eq. 8 was obtained and used further for calibration:

8 $$\:\frac{{I}_{HBRA}}{{I}_{CA}}=\text{exp}\left[-{\alpha\:}_{3}\:{\Delta{\dot{D}}}^{2}\text{exp}(-{\alpha\:}_{1}^{{\prime\:}}{\Delta\dot{D}})\right]\equiv\:RR$$

**Fig. 3:**
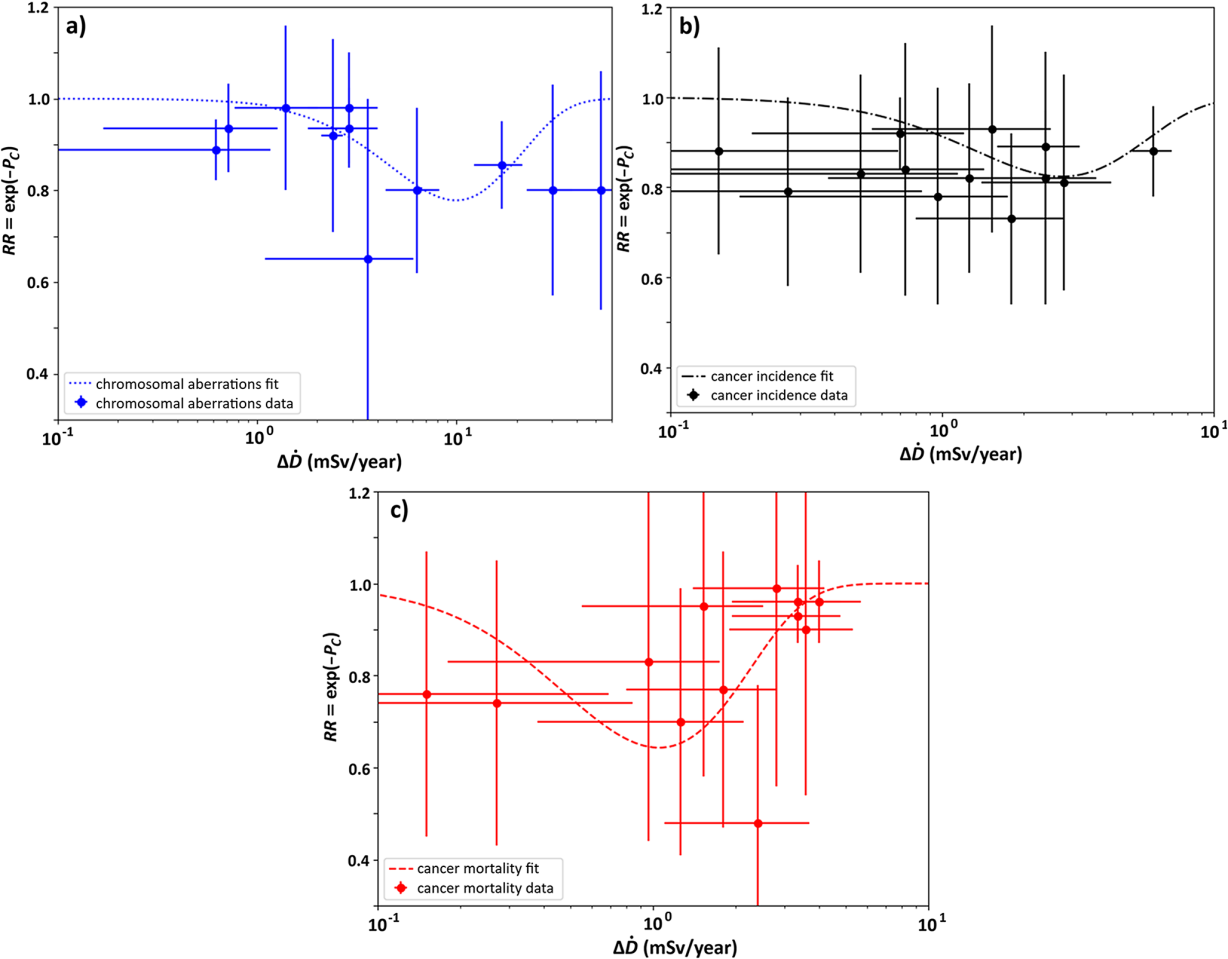
Dependence of relative risk (RR) from excess annual dose for: **a**) chromosomal aberrations: ${\alpha\:}_{3}$ = 0.019 ± 0.013 year^2^ • mSv^−2^, ${\alpha\:{\prime\:}}_{1}$ = 0.201 ± 0.054 year • mSv; **b**) cancer incidence : ${\alpha\:}_{3}$ = 0.188 ± 0.089 year^2^ • mSv^−2^, ${\alpha\:{\prime\:}}_{1}$ = 0.72 ± 0.17 year • mSv; **c**) cancer mortality : ${\alpha\:}_{3}$ = 3.0 ± 1.6 year^2^ • mSv^−2^, ${\alpha\:{\prime\:}}_{1}$ = 1.90 ± 0.29 year • mSv; *Each line represents the theoretical model fitted to the data points (model calibration) exhibiting the adaptive response effect. All uncertainties represent one standard deviation*

The calibration was performed using the ODR (Orthogonal Distance Regression) algorithm. The choice of the algorithm is motivated by the fact, that during curve fitting both horizontal and vertical uncertainties were taken into account, which was essential due to large uncertainties of both excess dose and relative risk. Calibration resulted in obtaining values of $\:{\alpha\:}_{1}^{{\prime\:}}$ and $\:{\alpha\:}_{3}$ what allowed plotting the fitted functions of relative risk $\:RR$ and probability of the adaptive response $\:{P}_{c}$. Obtained values of model’s parameters are presented in description of each figure (Fig. 3a and c) and in the Table 6.

Calculated parameters’ values were then used to plot the fitted curves described by equations 8 and 5 representing relative risk $\:RR$ and probability of radioadaptive response $\:{P}_{C}$, respectively. Plotted relationships (in semi-log scale) are presented as Fig. 3a, b and c. This means that the calculated parameters create a complete predictive model of adaptive response for constant dose-rate for chromosomal aberrations, cancer incidence and cancer mortality.

Values of maximum risk reduction were taken as $\:1-R{R}_{min}$, where $\:R{R}_{min}$ is the minimum of the fitted function.

**Table 6:** Values of adaptive response model’s parameters obtained by calibration for the case of chromosomal aberrations, cancer incidence and cancer mortality, see Fig. 3. All uncertainties represent one standard deviation

	Chromosomal aberrations	Cancer incidence	Cancer mortality
$\:{\alpha\:}_{3}$ (year^2^ • mSv^−2^)	0.019 ± 0.013	0.188 ± 0.089	3.0 ± 1.6
$\:{\alpha\:{\prime\:}}_{1}$ (year • mSv)	0.201 ± 0.054	0.72 ± 0.17	1.90 ± 0.29
Maximum risk reduction	22%	18%	36%

**Fig. 4:**
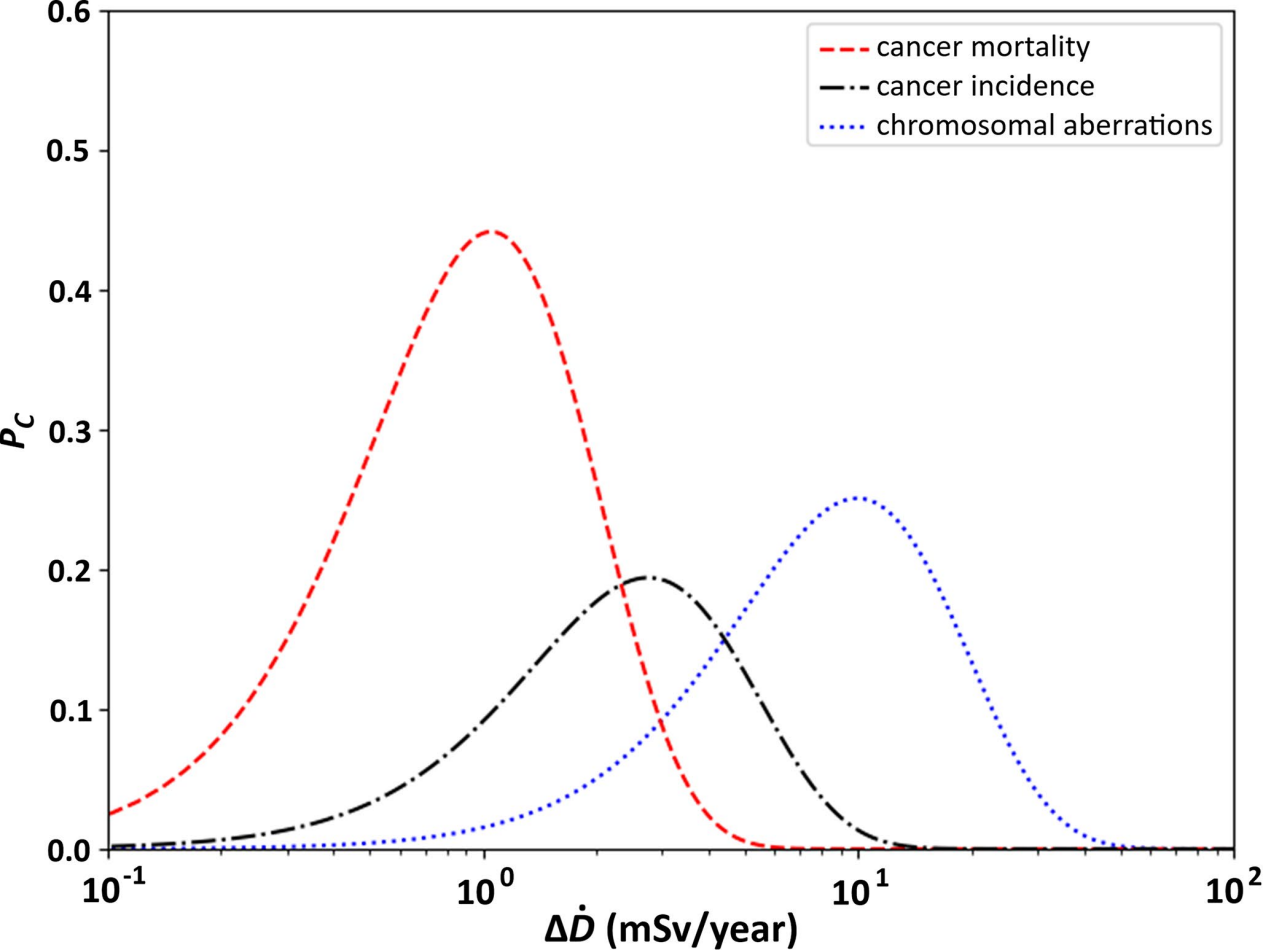
Comparison of calibrated model results of adaptive response probability function ($\:{P}_{C}$) for each of the three end-points: chromosomal aberrations (right peak), cancer incidence (middle peak) and cancer mortality (left peak)

For the sake of plot clarity, data with error bars and the fitted curve were presented on three separate plots, however in order to compare the calibrations for each of the end-points, all three curves were plotted on a single figure as the probability function of the adaptive response (Fig. 4).

Parameters obtained for the case of chromosomal aberrations allow for calculating the theoretical value of $\:\theta\:$ parameter that was used in the transition between Eqs. 1 and 4 – changing the model from single dose into its continuous form or to put it differently, from the form including doses as separate pulses to a form of dose-rate. That transition was described in Sect. 3, but for the sake of clarity it is once again presented below:

9 $$\:{\alpha\:}_{3}=2\beta\:\frac{{\alpha\:}_{0}{\theta\:}^{2}}{{\alpha\:}_{2}^{3}}$$

10 $$\:{\alpha\:}_{1}^{{\prime\:}}={\alpha\:}_{1}\theta\:$$

Both of those equations were discussed previously, so the following description will focus on parameters $\:\theta\:$ (describing saturation time of adaptive response) and $\:\beta\:$ (that is solely a scaling parameter currently bearing no physical meaning). Values of $\:{\alpha\:}_{3}$ and $\:{\alpha\:}_{1}^{{\prime\:}}$ were one of the objectives of this work and thus have been calculated. In turn, the values of $\:{\alpha\:}_{0}$, $\:{\alpha\:}_{1}$ and $\:{\alpha\:}_{2}$ were calculated for the case of in vitro Yonezawa-type irradiation in the previously cited paper (Fornalski et al. 2022a). Aside from broad and detailed description of the adaptive response phenomenon, the mentioned publication also presents results of calibration of the single dose(s) version of the same model as used here, but for the case of Raper-Yonezawa effect. Calculated values are: $\:{\alpha\:}_{0}={22.9}_{-4.0}^{+0.5}\:\text{G}{\text{y}}^{-2}{\text{h}}^{-3}$, $\:{\alpha\:}_{1}={79.4}_{-11.2}^{+5.5}\:\text{G}{\text{y}}^{-1}$ and $\:{\alpha\:}_{2}={0.0832}_{-0.0082}^{+0.0093}\:{\text{h}}^{-1}$. After converting the units from Gy to mSv (assuming radiation of weighing factor equal to 1 and uniform irradiation of the whole body) and from hours into years, values of $\:\beta\:$ and $\:\theta\:$ were calculated as: $\:\theta\:={2.53}_{-0.77}^{+0.70}$ year and $\:\beta\:{=0.037}_{-0.030}^{+0.028}$. Value of $\:\theta\:$ parameter may mean that after ∼ 28 months the probability of adaptive response for a person that moved from an area of normal (average) background radiation to HBRA saturates. In short, it may be assumed that the acclimatisation to elevated background radiation should take about 2.5 years. One should note, that this value is purely theoretical, was not confirmed by any study and is related only to adaptive response in relation to chromosomal aberrations in human peripheral blood lymphocytes. Because of lack of analogous data relating to cancer incidence and cancer mortality it was not possible to calculate the $\:\theta\:$ parameter for the two remaining cases.

## Discussion

### Dosimetry

The data points presented in Fig. 3, based on six different studies (see Table 3), use the relative values of dose-rate between HBRA and CA areas, $\:{\Delta\:}\dot{D}$. This approach avoids potential bias connected with dosimetric systems between all studies, as well as data collection methods. However, to have a complete view, the absolute value of $\:{\dot{D}}_{HBRA}$ was used for additional analysis. Such results are presented in Table 7. One has to note that those results are qualitatively the same as results from the main analysis (Table 6).

**Table 7:** *Values of adaptive response model’s parameters*,* analogical to ones presented in* table 6, *but here the dosimetry was based on*$\:{\dot{D}}_{HBRA}$, not $\:{\Delta\:}\dot{D}$. *All uncertainties represent one standard deviation*

	Chromosomal aberrations	Cancer incidence	Cancer mortality
$\:{\alpha\:}_{3}$ (year^2^ • mSv^−2^)	0.012 ± 0.006	0.058 ± 0.034	0.70 ± 0.29
$\:{\alpha\:{\prime\:}}_{1}$ (year • mSv)	0.171 ± 0.041	0.47 ± 0.15	1.06 ± 0.12
Maximum risk reduction	20%	13%	28%

### Detrimental effects

Considering the discrepancy of fitting results between methods (see for example, the large scatter of points in Fig. 2) and the large uncertainties (within one standard deviation) of calculated slope parameters of the LNT model by the ODR method ($\:a=0.25\pm\:0.20$ year/mSv), it was concluded that the collected data do not represent a linear correlation. Moreover, one should note that the area of uncertainty marked in Fig. 2 (Bayesian fit) indicates that the slope may be negative. This contradicts the LNT model assumptions, i.e. according to the UNSCEAR report, radiation risk for all solid cancer is usually estimated as 5%/Sv (UNSCEAR 2000). Thus, the detrimental data from Fig. 2 do not confirm the LNT model for radiation risk prediction in HBRAs. This conclusion is in line with findings presented in (Dobrzyński et al. 2015; David et al. 2021, 2022; Razavi-Toosi et al. 2023).

In order to confirm or unequivocally disprove the linear dependence of number of chromosomal aberrations on ionizing radiation dose one should collect a much larger set of applicable data or a dataset having smaller uncertainties (especially in the case where the value of uncertainty was greater than the value of the data). Moreover, fitting is largely affected by the rightmost point (Fig. 2). This point not only has the largest excess annual dose, but also a very small value of relative risk and uncertainty. No results of calibration and, more importantly, no standards or recommendations should be based on one data point, what further undermines the validity of the obtained linear fit.

### Radioadaptation effects

Figure 4 shows major differences between fitted curves for each of the dataset. This is an unexpected result. It was expected that minima of the curves representing relative risk $\:RR$ will be placed in the same range of dose-rate (or at least close to each other) with differing values of $\:RR$ associated with those extremums: the lowest $\:RR$ would be for cancer mortality, then cancer incidence and the most shallow one would be for chromosomal aberrations. This order was assumed, because it is known that there are many repair mechanisms present in the human body that should work on different stages of cancer initiation and development, beginning with damaged chromosome, mutation of the DNA and ending with the development of a neoplasm. It was considered that each stage should have its own part of defence mechanisms, that should overlap. Enhancing each part of defence mechanisms should then show the greatest reduction of relative risk at the end of the chain, not at its potential beginning.

First reason of such large difference between the fitted curves may be the issue of selecting members of study and control groups for the case of cancer mortality and incidence or more precisely the difference between selecting members for chromosomal aberration studies and the other two. Because of the fact that analyses of chromosomal aberrations are time-consuming and labour-intensive (taking and analysing blood sample from each person), the number of members in each group are typically limited to a few dozens or a few hundred people. Among the studies taken into account during the model calibration, the largest one consisted of 9997 persons in the control group and 17,837 total in the study groups (Ramachandran et al. 2013). In the case of cancer mortality and incidence, the smallest group was almost 50,000 (25258 in study group and 21837 in the control group) (Tao et al. 2000), however normally this number is even larger – up to several hundred thousand. The difference is due to the difficulties related to the analysis of chromosomal aberrations and simultaneous ease in obtaining large data sets for epidemiological studies. Usually, people chosen for chromosomal aberration studies are carefully selected in terms of received radiation doses so that differences between control and study group can be clearly defined.

**Fig. 5:**
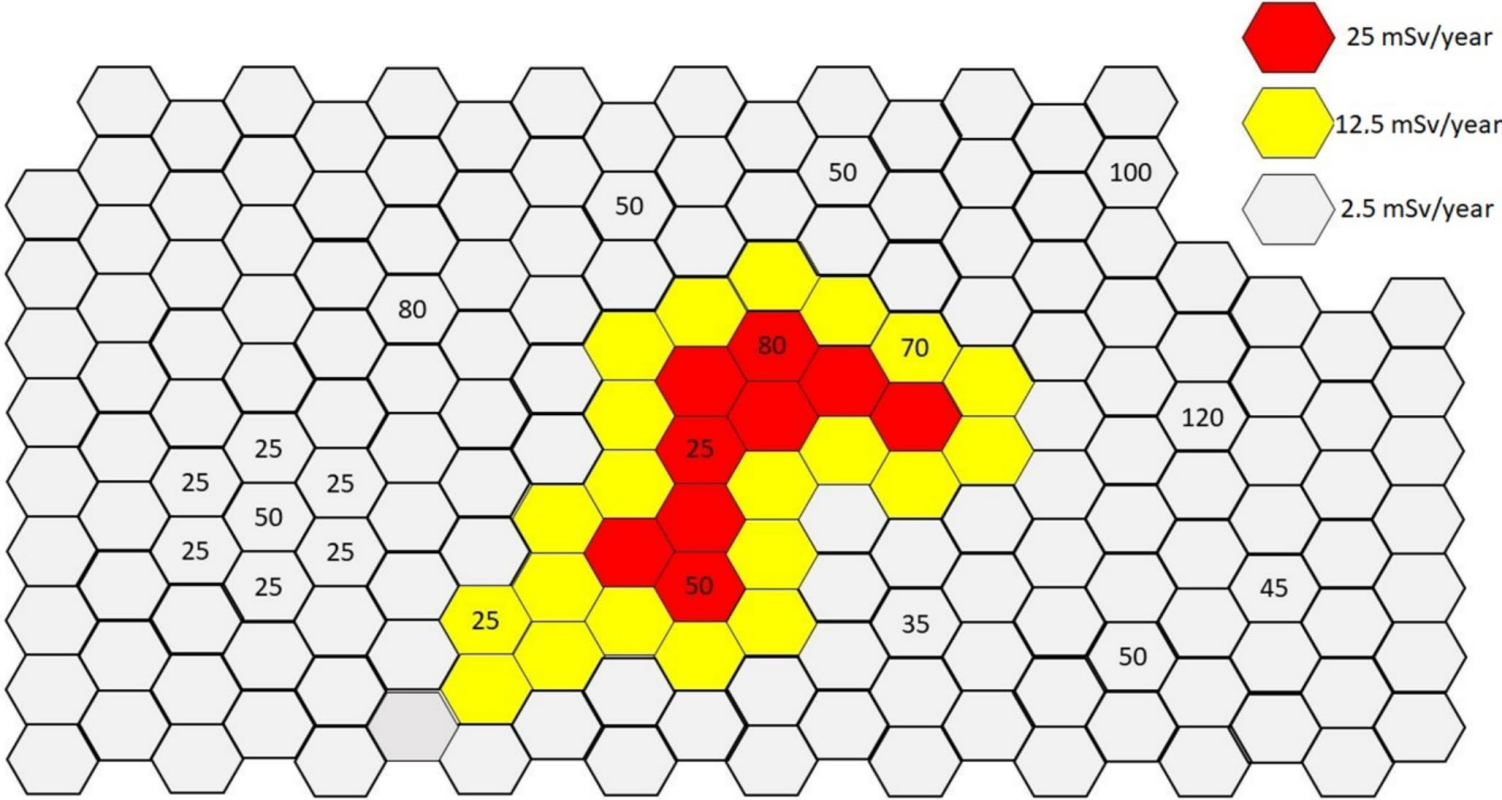
Schematic exemplary presentation of HBRA (yellow and red colours) against the control area (grey). Numbers inside each element represent population

HBRAs are a kind of anomaly. Their occurrence is rare and often limited to a small area affected by a series of phenomena of geological and meteorological nature. This in turn limits the elevated exposure to a certain, small area of land – most HBRAs are limited to an area smaller than the administrative region. If in the epidemiological studies, inhabitants of a whole region are taken as a study group, then the obtained effect of decrease or increase of e.g. cancer incidence will fade, and the results will be underestimated. Simultaneously, the excess dose estimated for the whole population will be reduced (the more people from outside the HBRA are taken into account, the lesser will be the overall dose). Figure 5 explains this idea more clearly.

Figure 5 schematically represents a simplified distribution of people in an exemplary region where numbers represent people living inside each element with different annual dose. In this example, number of HBRA inhabitants is 250 (whole region consists of 1000 people). Considering HBRA inhabitants as study group, their average annual dose is 20.25 mSv/year, while for control group it is 2.5 mSv/year. If the inhabitants of a whole region presented in Fig. 5 would be considered a study group, then the average annual dose would be 6.94 mSv/year. This simple example took into account a fourfold increase in the size of study group, but in reality sizes of study group in epidemiological studies may be dozen or more times larger than in the case of studies requiring collection of a biological sample. Then the effect of underestimating the annual dose may be even greater.

A second possible explanation of such significant discrepancy of results may be the outcome not only of different defence mechanisms functioning on different stages of development of DNA damage and formation of cancer, but also of different pathways involved. It is possible that different defence mechanisms and molecular pathways respond differently to the same values of doses (or dose-rates), even in the small dose range. For example, the mechanism of DNA repair may have a larger dose range tolerance than the mechanism responsible for the elimination of chromosomal aberrations, which results in differences between curves describing reduction of chromosomal aberration incidence and cancer incidence (Ananthanarayanan et al. 2023). Publication (Szumiel 2012) describes several, both positive and negative mechanisms of adaptive response. One of them is the stimulation of cell division, that on the one hand helps substitute the killed or damaged cells, but on the other, may accelerate cancer growth. Authors of (Wei and Sugahara 2000) provide a similar explanation. They suppose that the neoplasms are not a direct result of DNA damage, but of genomic instability. The genomic instability, aside from increased incidence of chromosomal aberrations and other genomic mutations describes also a series of phenomena causing cell (and its genetic material) division disorders. The issue of how these mechanisms work and their efficiency is strongly dependent on individual factors and is subject of many studies in the field of radiation biophysics and radiobiology (Tang and Loke 2015; Hussien 2023). It is the reason, among other things, that the contribution of individual radiosensitivity to the radioadaptive responses in HBRAs need to be further elucidated, as it is mechanistically (Averbeck et al. 2020) and epidemiologically (Berthel et al. 2019; Seibold et al. 2020; Gomolka et al. 2020; Rajaraman et al. 2018; Applegate et al. 2020) a complex matter. Ideally, molecular epidemiological studies would open good opportunities to model data of combined individual and epidemiological studies in order to assess radiation risks of HBRAs.

It should be noted that all results presented in this section are applicable to ∼ 45% of the human population (in Sect. 3 it was estimated that ∼ 2/5 of the studies regarding human chromosomes showed the existence of adaptive response) and thus should not be generally applied in radiation protection principles. Firstly, one should demonstrate that for each person subject to occupational exposure to ionizing radiation, the emergence of adaptive response is possible. Only occupational exposure is mentioned here, because (aside from purely scientific point of view) testing every person from the general public on their ability to manifest adaptive response, as it would not present a significant profit, but would only generate cost, would contradict the basic principle of radiation protection – the ALARA principle. However, even in the case of determining the individual radiosensitivity of each worker, their health should be subject to strict and constant monitoring programme, because the adaptive response may manifest at the level of chromosomal aberrations, but not further (e.g. cancer incidence). It may also change with time or future illnesses.

If one were to apply the value of $\:\theta\:$ parameter to persons subject to occupational exposure, it should be taken into account that (in theory) such people would be exposed to elevated levels of ionizing radiation (at most) during only 8 out of 24 h. One might say, that the time of saturation of adaptive response probability for them would then be four times longer, than for HBRA inhabitants. However, the human organism is a complex system and as such, its response should not be linear. Another group, that should be mentioned in terms of adaptive response are astronauts (Fornalski 2022). During their space missions they are not only exposed to constantly elevated dose-rates, but also to additional pulses of ionising radiation originating from solar flares of different radiation qualities. Indeed, astronauts may be exposed to different radiation qualities at the same time and varying doses and dose-rates. The opportunity of studying adaptive response among astronauts would provide an invaluable chance (especially for the strict selection process of the candidates) to study a combined approach to adaptive response, i.e. the combination of constantly elevated dose rate and the Raper-Yonezawa effect (Mortazavi et al. 2003b).

## Conclusions

This paper raises the topic of radiation adaptive response in several different HBRAs concerning chromosomal aberrations in human lymphocytes, cancer incidence (other than leukaemia) and cancer mortality. A model is presented describing the dependence of adaptive response probability function on annual dose and calibrated for the three end-points. Calibration was performed using dedicated ODR (orthogonal distance regression) algorithm. The results of calibration consist of plots depicting the dependence of relative risk and probability of adaptive response on excess annual dose of ionizing radiation and values of parameters calculated for each curve. Results, along with the theoretical value of $\:\theta\:$ parameter (time required for the adaptive response probability to saturate, or simplifying a bit – time needed for acclimatization to elevated levels of background radiation), were described in Sect. 5, whereas they were discussed in Sect. 6. The theoretical “acclimatization” time $\:\theta\:$ is ∼ 2.5 years. One should remember that this value is purely theoretical and has been calculated by comparing two calibrations of the same model, one for the case of constant exposure and the second for fractioned dose in the Raper-Yonezawa scheme (two doses). The first dataset concerned lymphocytes in the blood samples of HBRA inhabitants (in vivo irradiation), while the second one was related to in vitro studies, where the blood samples were irradiated after drawing the sample. Unfortunately, the uncertainties of calculated parameters are large (in some cases amount to up to 50% of the parameter’s value), which may lead one to doubt the obtained results. Surely the uncertainties as well as result discrepancies could be reduced if the datasets were larger, though at this moment these were not available. The number of appropriate data points used were those that provided sufficient information on incidence of one of the end-points (chromosomal aberrations, cancer incidence or cancer mortality) among the inhabitants of HBRA and CA (or the relative risk) and average annual doses for each of the groups. Conducting more studies that meet the aforementioned requirements would allow to get more reliable results. In the case of chromosomal aberrations this would require more costly and time-consuming studies of the inhabitants of remote corners of the world. Theoretically, it would be easier to perform more epidemiological studies, but these would need a thorough knowledge of the region or country or broad demographic and economic studies in order to find also appropriate control areas. Another solution would be to limit epidemiological studies to people living in the “strict” HBRA, but this would require a precise background interview of each person (especially estimations of the annual doses, their medical histories and habits, e.g. tobacco use). It should be noted, that the results and conclusions described in this paper regarding radiation adaptive response concern ∼ 45% of the population, thus should not be generally applied to radiation protection. The aim of this work was not to prove the universality or existence of radiation adaptive response, but only to model the probability function of its occurrence in relation to ionizing radiation dose in the case if it is present – which is not always the case.

## Electronic supplementary material

Below is the link to the electronic supplementary material.

Supplementary Material 1

## Data Availability

No datasets were generated or analysed during the current study.

## References

[CR57] Ananthanarayanan AT, Raavi V, Srinivas-Kondaveeti S, Ramachandran I, Perumal V (2023) Insights on the radiation-induced adaptive response at the cellular level and its implications in cancer therapy. Cytogenet Genome Res. 10.1159/000534500. Epub ahead of print. PMID: 37906989 10.1159/000534500 37906989

[CR1] Applegate KE, Rühm W, Wojcik A, Bourguignon M, Brenner A, Hamasaki K, Imai T, Imaizumi M, Imaoka T, Kakinuma S, Kamada T, Nishimura N, Okonogi N, Ozasa K, Rübe CE, Sadakane A, Sakata R, Shimada Y, Yoshida K, Bouffler S (2020) Individual response of humans to ionising radiation: governing factors and importance for radiological protection. Radiat Environ Biophys 59(2):185–209. 10.1007/s00411-020-00837-y 32146555 10.1007/s00411-020-00837-y

[CR3] Averbeck D (2023) Low-dose Non-targeted effects and mitochondrial control. Int J Mol Sci 24:11460. 10.3390/ijms241411460 37511215 10.3390/ijms241411460 PMC10380638

[CR2] Averbeck D, Candéias S, Chandna S, Foray N, Friedl AA, Haghdoost S, Jeggo PA, Lumniczky K, Paris F, Quintens R, Sabatier L (2020) Establishing mechanisms affecting the individual response to ionizing radiation. Int J Radiat Biol 96(3):297–323. 10.1080/09553002.2019.1704908 31852363 10.1080/09553002.2019.1704908

[CR4] Berthel E, Foray N, Ferlazzo ML (2019) The nucleoshuttling of the ATM protein: a unified model to describe the individual response to high- and low-dose of radiation? Cancers 11:905 31261657 10.3390/cancers11070905 PMC6678722

[CR5] Bravard A, Luccioni C, Moustacchi E, Rigaud O (1999) Contribution of antioxidant enzymes to the adaptive response to ionizing radiation of human lymphoblasts. Int J Radiat Biol 75:639–645 10374946 10.1080/095530099140285

[CR6] Chen D, Wei L (1991) Chromosome aberration, Cancer Mortality and Hormetic Phenomena among inhabitants in Areas of high background Radiation in China. J Radiation Res (32) Supplement 2: 46–53 1823366

[CR7] Cheryian VD, Kurien CJ, Das B, Ramachandran EN, Karuppasamy CV, Thampi MV, George KP, Kesavan PC, Koya PKM (1999) Genetic monitoring of the Human Population from High-Level Natural Radiation areas of Kerala on the Southwest Coast of India. II. Incidence of Numerical and Structural chromosomal aberrations in the lymphocytes of newborns. Radiat Res (152): 154–S158 10564959

[CR8] Darby S, Hill D, Auvinen A, Barros-Dios JM, Baysson H, Bochicchio F, Deo H, Falk R, Forastiere F, Hakama M, Heid I, Kreienbrock L, Kreuzer M, Lagarde F, Mäkeläinen I, Muirhead C, Oberaigner W, Pershagen G, Ruano-Ravina A, Ruosteenoja E, Rosario AS, Tirmarche M, Tomášek L, Whitley E, Wichmann HE, Doll R (2005) Radon in homes and risk of lung cancer: collaborative analysis of individual data from 13 European case-control studies. BMJ 330(7485):223 15613366 10.1136/bmj.38308.477650.63 PMC546066

[CR10] David E, Wolfson M, Fraifeld VE (2021) Background radiation impacts human longevity and cancer mortality: reconsidering the linear no-threshold paradigm. Biogerontology 22:189–195. 10.1007/s10522-020-09909-4 33479810 10.1007/s10522-020-09909-4

[CR9] David E, Bitan R, Atlas S, Wolfson M, Fraifeld VE (2022) Correlative links between natural radiation and life expectancy in the US population. Biogerontology 23:425–430. 10.1007/s10522-022-09971-0 35727470 10.1007/s10522-022-09971-0

[CR11] Devic C, Ferlazzo ML, Berthel E, Foray N (2020) Influence of individual radiosensitivity on the hormesis phenomenon: toward a mechanistic explanation based on the nucleoshuttling of ATM protein. Dose Response 18:1559325820913784 32425719 10.1177/1559325820913784 PMC7218313

[CR12] Dimova EG, Bryant PE, Chankova SG (2008) Adaptive response: some underlying mechanisms and open questions. Genet Mol Biol 31(2)

[CR14] Dobrzyński L, Fornalski KW, Feinendegen LE (2015) Cancer Mortality among people living in Areas with various levels of natural background Radiation. Dose Response 13(3) 10.1177/1559325815592391 PMC4674188 26674931

[CR13] Dobrzyński L, Fornalski KW, Reszczyńska J (2018) Meta-analysis of thirty two case-control and two ecological radon studies of lung cancer. J Radiat Res 59(2):149–163 29186473 10.1093/jrr/rrx061 PMC5950923

[CR15] Dobrzyński L, Fornalski KW, Reszczyńska J, Janiak M (2019) Modeling cell reactions to Ionizing Radiation: from a lesion to a Cancer. Dose-Response 17(2) 10.1177/1559325819838434 PMC6454661 31001068

[CR16] Fornalski KW (2015) Applications of the robust Bayesian regression analysis. Int J Soc Syst Sci 7(4):314–333. 10.1504/IJSSS.2015.073223

[CR17] Fornalski KW (2022) Radioadaptation and radioresistance during deep space travels. J Space Saf Eng 9385–389. 10.1016/j.jsse.2022.04.001

[CR20] Fornalski KW, Adamowski Ł, Dobrzyński L, Jarmakiewicz R, Powojska A, Reszczyńska J (2022a) The radiation adaptive response and priming dose influence: the quantification of the Raper-Yonezawa effect and its three-parameter model for postradiation DNA lesions and mutations. Radiat Environ Biophys 61(7):221–239 10.1007/s00411-022-00963-9 PMC9021059 35150289

[CR18] Fornalski KW, Adamowski Ł, Bugała E, Jarmakiewicz R, Kirejczyk M, Kopyciński J, Krasowska J, Kukulski P, Piotrowski Ł, Ponikowska J, Reszczyńska J, Słonecka I, Wysocki P, Dobrzyński L (2022b) Biophysical modeling of the ionizing radiation influence on cells using the stochastic (Monte Carlo) and deterministic (analytical) approaches. Dose-Response, 20(4). 10.1177/15593258221138506 10.1177/15593258221138506 PMC9706082 36458282

[CR19] Fornalski KW, Adamowski Ł, Bugała E, Jarmakiewicz R, Krasowska J, Piotrowski Ł (2024) Radiation adaptive response: the biophysical phenomenon and its theoretical description. Radiation Protection Dosimetry (accepted for publication), 10.1093/rpd/ncae053 10.1093/rpd/ncae053 39540508

[CR21] Ghiassi-Nejad M, Zakeri F, Assaei RG, Kariminia A (2004) Long-term immune and cytogenetic effects of high level natural radiation on Ramsar inhabitants in Iran. J Environ Radioact (74):107–116 10.1016/j.jenvrad.2003.12.001 15063540

[CR22] Gomolka M, Blyth B, Bourguignon M, Badie C, Schmitz A, Talbot C, Hoeschen C, Salomaa S (2020) Potential screening assays for individual radiation sensitivity and susceptibility and their current validation state. Int J Radiat Biol 96(3):280–296. 10.1080/09553002.2019.1642544 31347938 10.1080/09553002.2019.1642544

[CR23] Guéguen Y, Bontemps A, Ebrahimian TG (2019) Adaptive responses to low doses of radiation or chemicals: their cellular and molecular mechanisms. Cell Mol Life Sci (76):1255–1273 10.1007/s00018-018-2987-5 PMC11105647 30535789

[CR24] Hayata I, Wang C, Zhang W, Chen D, Minamihisamatsu M, Morishima H, Yuan Y, Wei L, Sugahara T (2000) Chromosome translocation in residents of the high background Radiation areas in Southern China. J Radiat Res 41(supplement):69–74 11142214 10.1269/jrr.41.s69

[CR26] HBRRG (High Background Radiation Research Group, China) (1980) Health Survey in High Background Radiation Areas in China, Science, Vol. 209 7403855

[CR25] Hendry JH, Simon SL, Wojcik A, Sohrabi M, Burkart W, Cardis E, Laurier D, Tirmarche M, Hayata I (2009) Human exposure to high natural background radiation: what can it teach us about radiation risks? J Radiol Prot 29:A29–A42. 10.1088/0952-4746/29/2A/S03 19454802 10.1088/0952-4746/29/2A/S03 PMC4030667

[CR27] Hussien SM (2023) Radio-adaptive Response Induced by low-dose Ionizing Radiation in Innate Immunity for Radiotherapy. Health Phys 124(3):166–174 36719932 10.1097/HP.0000000000001649

[CR28] Jain V, Saini D, Kumar PRV, Jaikrishan G, Das B (2017) Efficient repair of DNA double strand breaks in individuals from high level natural radiation areas of Kerala coast, south-west India. Mutat Res 806:39–50 28963924 10.1016/j.mrfmmm.2017.09.003

[CR29] Jiang T, Hayata I, Wang C, Nakai S, Yao S, Yuan Y, Dai L, Liu Q, Chen D, Wei L, Sugahara T (2000) Dose-effect relationship of Dicentric and Ring chromosomes in lymphocytes of individuals living in the high background Radiation areas in China. J Radiat Res 41(Supplement):63–68 11142213 10.1269/jrr.41.s63

[CR30] Kendall GM, Little MP, Wakeford R, Bunch KJ, Miles JCH, Vincent TJ, Meara JR, Murphy MFG (2013) A record-based case-control study of natural background radiation and the incidence of childhood leukaemia and other cancers in Great Britain during 1980–2006. Leukemia 27(1):3–9 22766784 10.1038/leu.2012.151 PMC3998763

[CR31] Lall R, Ganapathy S, Yang M, Xiao S, Xu T, Su H, Shadfan M, Asara JM, Ha CS, Ben-Sahra I et al (2014) Low-dose radiation exposure induces a HIF-1-mediated adaptive and protective metabolic response. Cell Death Differ 21:836–844 24583639 10.1038/cdd.2014.24 PMC3978308

[CR32] Laurier D, Billarand Y, Klokov D, Lauraud K (2023) The scientific basis for the use of the linear no-threshold (LNT) model at low doses and dose rates in radiological protection. J Radiol Prot 43(2) 10.1088/1361-6498/acdfd7 37339605

[CR33] Lubin JH, Boice JD (1997) Lung Cancer Risk from Residential Radon: Meta-analysis of eight epidemiologic studies. JNCI: J Natl Cancer Inst 89(1):49–57 10.1093/jnci/89.1.49 8978406

[CR34] Matsumoto H, Takahashi A, Ohnishi T (2007) Nitric oxide radicals choreograph a radioadaptive response. Cancer Res 67:8574–8579 17875696 10.1158/0008-5472.CAN-07-1913

[CR35] Mazzei-Abba A, Folly CL, Kreis C, Ammann RA, Adam C, Brack E, Egger M, Kuehni CE, Spycher BD (2021) External background ionizing radiation and childhood cancer: update of a nationwide cohort analysis. J Environ Radioact 238–239:106734 10.1016/j.jenvrad.2021.106734 34521026

[CR36] Mohammadi S, Taghavi-Dehaghani M, Gharaati MR, Masoomi R, Ghiassi-Nejad M (2006) Adaptive response of blood lymphocytes of inhabitants residing in high background radiation areas of ramsar- micronuclei, apoptosis and comet assays. J Radiat Res 47(3–4):279–285. 10.1269/jrr.0575 16988494 10.1269/jrr.0575

[CR38] Mortazavi SMJ, Ghiassi-Nejad M, Ikushima T, Assaie R, Heidary A, Varzegar R, Zakeri F, Asghari K, Esmaili A (2003a) Are the inhabitants of high background Radiation areas of Ramsar more Radioresistant? Scope of the Problem and the need for Future studies. Iran J Radiol 1:1–2

[CR39] Mortazavi SMJ, Cameron J, Niroomand-rad A (2003b) Adaptive response studies may help choose astronauts for long-term space travel. Adv Space Res 31(6):1543–1551 12971409 10.1016/s0273-1177(03)00089-9

[CR37] Mortazavi SMJ, Mortazavi G, Mortazavi SAR, Paknahad M (2019) Is induction of anomalies in lymphocytes of the residents of high background Radiation Areas Associated with increased Cancer risk? J Biomed Phys Eng 9(3):367–372. 10.31661/jbpe.v9i3Jun.654 31341882 10.31661/jbpe.v9i3Jun.654 PMC6613150

[CR40] Nair MK, Nambi KSV, Amma NS, Gangadharan P, Jayalekshmi P, Jayadevan S, Cherian V, Nair-Reghuram K (1999) Population Study in the high natural background Radiation Area in Kerala, India. Radiat Res (152):145–148 10564957

[CR41] Nair RRK, Rajan B, Akiba S, Jayalekshmi P, Nair MK, Gangadharan P, Koga T, Morishima H, Nakamura S, Sugahara T (2009) Background radiation and cancer incidence in Kerala, India – Karungappally cohort study. Health Physics (96) 10.1097/01.HP.0000327646.54923.11 19066487

[CR42] Paraswani N, Thoh M, Bhilwade HN, Ghosh A (2018) Early antioxidant responses via the concerted activation of NF-_B and Nrf2 characterize the gamma-radiation-induced adaptive response in quiescent human peripheral blood mononuclear cells. Mutat Res Genet Toxicol Environ Mutagen 831:50–61 29875077 10.1016/j.mrgentox.2018.04.007

[CR43] Rajaraman P, Hauptmann M, Bouffler S, Wojcik A (2018) Human individual radiation sensitivity and prospects for prediction. Ann ICRP 47(3–4):126–141. 10.1177/0146645318764091 29648458 10.1177/0146645318764091

[CR44] Ramachandran E, Karuppasamy C, Cheriyan V, Soren D, Das B, Anilkumar V, Koya PKM, Seshadri M (2013) Cytogenetic studies on newborns from high and normal level natural radiation areas of Kerala in southwest coast of India. Int J Radiat Biol (89):259–267 10.3109/09553002.2013.747014 23134065

[CR45] Ramachandran EN, Karuppasamy CV, Kumar VA, Soren DC, Kumar PRV, Koya PKM, Jaikrishan G, Das B (2017) Radio-adaptive response in peripheral blood lymphocytes of individuals residing in high-level natural radiation areas of Kerala in the southwest coast of India. Mutagenesis 32(2):267–273. 10.1093/mutage/gew057 27831478 10.1093/mutage/gew057

[CR46] Razavi-Toosi SMT, Bakshi M, Yousefi R (2023) Assessing cancer mortality in high natural background radiation areas: a systematic review. J Curr Oncol Med Sci 3(3):532–540

[CR47] Seibold P, Auvinen A, Averbeck D, Bourguignon M, Hartikainen JM, Hoeschen C, Laurent O, Noël G, Sabatier L, Salomaa S, Blettner M (2020) Clinical and epidemiological observations on individual radiation sensitivity and susceptibility. Int J Radiat Biol 96(3):324–339. 10.1080/09553002.2019.1665209 31539290 10.1080/09553002.2019.1665209

[CR48] Shimura N, Kojima S (2018) The Lowest Radiation Dose having molecular changes in the living body. Dose Response 16(2):1559325818777326. 10.1177/1559325818777326 29977175 10.1177/1559325818777326 PMC6024299

[CR49] Spycher BD, Lupatsch JE, Zwahlen M, Roosli M, Niggli F, Grotzer MA, Rischewski J, Egger M, Kuehli CE (2015) Background Ionizing Radiation and the risk of Childhood Cancer: a Census-based Nationwide Cohort Study. Environmental Health Perspectives, 123(6):622–628. 10.1289/ehp.1408548 PMC4455589 25707026

[CR50] Su S, Zhou S, Wen C, Zou J, Zhang D, Geng J, Yang M, Liu M, Li L, Wen W (2018) Evidence for adaptive response in a molecular epidemiological study of the inhabitants of a high background-radiation area of Yangjiang, China. Health Phys 115:227–234 29957687 10.1097/HP.0000000000000860

[CR51] Syaifudin M, Purnami S, Rahardjo T, Kurnia I, Rahajeng N, Nurhayati SD, Mailana W, Ramadhani D, Agesti-Suvifan V, Kisnanto T, Pudjadi E (2018) Cytogenetic and molecular damages in blood lymphocyte of inhabitants living in high Level Natural Radiation Area (HLNRA) of Botteng Village, Mamuju, West Sulawesi, Indonesia. Radiation Environ Med. 7(2):65–76

[CR52] Szumiel I (2012) Hormeza popromienna: autofagia i inne mechanizmy komórkowe (English: Radiation hormesis: autophagy and other cellular mechanisms). Postępy Techniki Jądrowej 55(1):7–15

[CR53] Talebian H, Monfared AS, Niaki HA, Fattahi S, Bakhtiari E, Changizi V (2020) Investigating the expression level of NF-KB and HIF1A genes among the inhabitants of two different background radiation areas in Ramsar, Iran. J Environ Radioact 220–221:106292. 10.1016/j.jenvrad.2020.106292 10.1016/j.jenvrad.2020.106292 32658641

[CR54] Tang FR, Loke WK (2015) Molecular mechanisms of low dose ionizing radiation-induced hormesis, adaptive responses, radioresistance, bystander effects, and genomic instability. Int J Radiat Biol 91(1):13–27. 10.3109/09553002.2014.937510 24975555 10.3109/09553002.2014.937510

[CR55] Tao Z, Zha Y, Akiba S, Sun Q, Zou J, Li J, Liu Y, Kato H, Sugahara T, Wei L (2000) Cancer Mortality in the high background Radiation areas of Yangjiang, China during the period between 1979 and 1995. J Radiat Res 41(SUPPL):31–41 11142210 10.1269/jrr.41.s31

[CR56] Tapio S, Jacob V (2007) Radioadaptive response revisited. Radiat Environ Biophys 46(1):1–12 17131131 10.1007/s00411-006-0078-8

[CR58] Toledo SM, Asaad N, Venkatachalam P, Li L, Howell RW, Spitz DR, Azzam EI (2006) Adaptive responses to low-dose/low dose-rate gamma rays in normal human fibroblasts: the role of growth architecture and oxidative metabolism. Radiat Res 166:849–857 17149977 10.1667/RR0640.1

[CR59] UNSCEAR 1994 (United Nations Scientific Committee on the Effects of Atomic Radiation). Sources and effects of ionizing radiation. Annex B: Adaptive responses to radiation in cells and organisms. UNSCEAR Report (1994)

[CR60] UNSCEAR 2000 (United Nations Scientific Committee on the Effects of Atomic Radiation) report 2000. Sources and Effects of Ionizing Radiation, UNSCEAR 2000 Report to the General Assembly, with Scientific Annexes

[CR61] UNSCEAR 2008 (United Nations Scientific Committee on the Effects of Atomic Radiation) (2008) 2008 report, annex B: exposures of the public and workers from various sources of radiation. UNSCEAR Rep

[CR62] UNSCEAR 2021 (United Nations Scientific Committee on the Effects of Atomic Radiation) III - sources, effects and risks of ionizing radiation. Annex C: Biological mechanisms relevant for the inference of cancer risks from low-dose and low-dose-rate radiation. Section III.F.4 - adaptive response. UNSCEAR Report (2020/2021)

[CR63] Wang Z, Boice JDJr, Wei L, Beebe GW, Zha Y, Kaplan MM, Tao Z, Maxon HR III, Zhang S, Schneider AB, Tan B, Wesseler TA, Chen D, Ershow AG, Kleinerman RA, Littlefield LG, Preston D (1990) Thyroid nodularity and chromosome aberrations among women in areas of high background Radiation in China. J Natl Cancer Inst (82), 6 10.1093/jnci/82.6.478 2313719

[CR64] Wei L, Sugahara T (2000) An introductory overview of the Epidemiological Study on the Population at the high background Radiation areas in Yangjiang, China. J Radiat Res 41:SUPPL 10.1269/jrr.41.s1 11142208

[CR65] Wei L, Zha Y, Tao Z, He W, Chen D, Yuan Y (1990) Epidemiological investigation in high background radiation areas of Yangjiang, China. International Conference on High Levels of Natural Radiation, Ramsar, 3–7 Nov. 1990

[CR66] Wojcik A, Zölzer F (2024) The scientific nature of the linear no-threshold (LNT) model used in the system of radiological protection. Radiat Environ Biophys. 10.1007/s00411-024-01092-1 10.1007/s00411-024-01092-1 PMC11588861 39222266

[CR67] Zakeri F, Rajabpour MR, Haeri SA, Kanda R, Hayata I, Nakamura S, Sugahara T, Ahmadpour MJ (2011) Chromosome aberrations in peripheral blood lymphocytes of individuals living in high background radiation areas of Ramsar, Iran. Radiat Environ Biophys 50:571–578 21894441 10.1007/s00411-011-0381-x

[CR69] Zhang W, Wang C, Chen D, Minamihisamatsu M, Morishima H, Yuan Y, Wie L, Sugahara T, Hayata I (2003) Imperceptible effect of Radiation based on stable type chromosome aberrations accumulated in the lymphocytes of residents in the high background Radiation Area in China. J Radiat Res 44:69–74 12841602 10.1269/jrr.44.69

[CR68] Zhang W, Wang C, Chen D, Minamihisamatsu M, Morishima H, Yuan Y, Wei L, Sugahara T, Hayata I (2004) Effects of Smoking on chromosomes compared with that of Radiation in the residents of a high-background Radiation Area in China. J Radiat Res 45:441–446 15613790 10.1269/jrr.45.441

