# Supplementary material for: Radiation adaptive response for constant dose-rate irradiation in high background radiation areas
Source: Radiat Environ Biophys. 2024 Oct 29;64(1):29–44. doi: 10.1007/s00411-024-01093-0 (PMC11971215; doi:10.1007/s00411-024-01093-0)
Supplement: Supplementary file 1 — Supplementary Material 1 [file 411_2024_1093_MOESM1_ESM.pdf]

SUPPLEMENTARY MATERIAL TO THE PAPER:

Bugała E., Fornalski K.W. *Radiation adaptive response for constant dose-rate irradiation in high background radiation areas*. Radiation and Environmental Biophysics, 2024

Part A

Data disproving the adaptive response

Ghiassi-Nejad et al. 2003 [1]

Publication describes studies conducted among 80 inhabitants from the Ramsar region in Iran: 50 people from the HBRA and 30 from the control area. Average annual doses were 13±12 mSv and 2.30(9) mSv respectively. Results presented in this work include number of analysed cells and the number of observed chromosomal aberrations with classification into translocations, inversions, deletions and other. In the HBRA group the number of observed aberrations was 307 in 5122 cells ($I_{HBRA} = 0.0599 \pm 0.0035$), while in the control group it was 46 aberrations in 3054 cells ($I_{CA} = 0.0151 \pm 0.0022$). Relative risk is thus $RR = 3.98 \pm 0.64$. Annual excess dose $\Delta\dot{D} = 11 \pm 12$ mSv (value of uncertainty is not a result of an error, but of a significant uncertainty of HBRA dose measurement).

Chen and Wei 1991 [2]

This publication describes similar study to the one described above. Control group consisted of 104 persons, while study group was divided in two – a group of 106 and 120 persons. Annual doses in each group were presented as an interval: 0.70-0.79 mGy, 2.00-2.19 mGy and 2.60-2.80 mGy respectively. For the purpose of this work, the centre value of each interval has been assumed as the annual dose for each group, then the doses are: 0.75(19) mGy, 2.1(52) mGy and 2.70(68) mGy. Uncertainties were assumed as 25% of the dose value. It should be noted that the calculated uncertainties are greater than the width of each interval, so even if the average dose for each interval is different than calculated, it would still be covered by the uncertainty. Results regarding incidence of chromosomal aberrations were divided into two categories: dicentrics + rings and translocations + inversions. Thanks to the fact that the authors provided the number of cells studied in each group it was possible to recalculate the number of detected aberrations in each group and obtain the incidence of all aberrations collectively. The risk ration for each of the study groups were $RR = 4.8 \pm 5.4$ for the low dose group and $RR = 9 \pm 22$ for the high dose group. Uncertainty values were calculated in line with the propagation of uncertainty. The fact that the uncertainty is greater that its corresponding value stems from the value of aberration incidence in the control group being equal to the value of its uncertainty.

Jiang et al. 2000 [3]

This study was conducted among 39 inhabitants of China, of which 22 persons living in the HBRA while the remaining 17 – in the control area. Data presented in the publication consisted of each person's age, annual dose, calculated lifetime dose (up to the moment of study), number of cells studies and the number of detected aberrations per 1000 cells, along with uncertainty. For each

group the average annual dose and aberration incidence were calculated (both of them with one standard deviation uncertainty). The calculated relative risk was $RR = 1.24 \pm 0.70$ and average annual excess dose $\Delta\dot{D} = 1.99 \pm 0.33$ mSv.

Wang et al. 1990 [4]

The following publication describes studies conducted in China on the influence of elevated levels of background radiation on the incidence of thyroid nodules and chromosomal aberrations in lymphocytes. For the purpose of this paper, only the latter aspect of the cited publication was taken into account. Study considered 200 women – 100 from the control area, the other half from the HBRA. Average annual dose was 2.89±0.72 mSv in the HBRA and 1.00±0.25 mSv in the control area. Uncertainty of doses were assumed as 25% of the dose values. Annual excess dose was thus $\Delta\dot{D} = 1.9 \pm 1.0$ mSv (uncertainty calculated in line with the propagation of uncertainty). Aberration frequencies per 100 cells were $I_{CA} = 0.23 \pm 0.05$ for the control area and $I_{HBRA} = 0.44 \pm 0.07$ for the HBRA. Relative risk was $RR = 1.91 \pm 0.52$.

Zakeri et al. 2011 [5]

Next publication describes study conducted among 25 women aged 50 to 63 years living in Ramsar region in Iran. 15 of them were HBRA inhabitants, other 10 were assumed as the control group. The paper provided information on each of the women separately regarding their age, calculated lifetime doses, number of detected aberrations and number of cells studied. Doses were calculated based on two methods of measurement: electronic pocket dosimeter (EPD) and optically stimulated luminescence dosimeter (OSLD). For the purpose of having one dose value for each person (and later for the whole group – CA and HBRA) an arithmetic mean was calculated from the two measured dose values. Then the mean and standard deviation for each group were calculated. Average number of chromosomal aberrations and its uncertainty were included in the publication. Results concerning excess annual dose and relative risk were $\Delta\dot{D} = 3.1 \pm 2.0$ mSv and $RR = 3.7 \pm 3.4$.

Zhang et al. 2003 [6]

Last publication describes studies regarding children (aged 10-14 years) and elderly persons (aged 53-89 years) living in China. Study group consisted of 21 persons (6 children and 15 elders), while the control group was 19 persons (8 children and 11 elders). This paper provides information on sex, age, number of cells studied and detected aberrations, incidence of aberrations per 1000 cells and estimated annual and lifetime dose for each of the persons studied. Data regarding aberration incidence and annual doses were averaged for each of the groups (HBRA and control group). Uncertainties were calculated as one standard deviation. Results were $\Delta\dot{D} = 2.07 \pm 0.34$ mSv and $RR = 1.18 \pm 0.99$.

Part B

Adaptive response data used for calibration

High Background Radiation Research Group, China 1980 [7]

Paper describes studies conducted among inhabitants of two HBRA in the Yangjiang region of Guangdong province in China, where the natural background radiation is 2-3 times higher than in the surrounding regions. According to authors 90.6% of the population has lived in the region for at least 6 generations. Studied HBRA were paired with similar (geographically and demographically) control areas. Publication provides information regarding annual effective dose in the HBRA and CA, as well as number of detected chromosomal aberrations (without differentiating its category) per 100 cells. Additionally total number of detected aberrations and number of studied persons (106 from the HBRA and 104 from the CA) are given. Whole body effective dose were 231 mrem (2.31 mSv) and 96 mrem (0.96 mSv). Dose uncertainty was assumed to be 25% of the dose value, thus $\Delta\dot{D} = 1.39 \pm 0.35$ mSv (calculated in line with uncertainty propagation). Calculated aberration incidence were $I_{HBRA} = 0.270 \pm 0.036$ and $I_{CA} = 0.274 \pm 0.036$, thus relative risk was $RR = 0.98 \pm 0.18$.

Hayata et al. 2000 [8]

This paper is focused on one particular type of chromosomal aberrations – translocation among inhabitants of HBRA located in southern China. The study covered a total of 17 persons: 9 from the HBRA and 8 from the CA. Number of detected translocations, number of studied cells and translocation incidence per 1000 cells are given for each of them. Publication also provides information on annual absorbed dose calculated (based on measured kerma) for each person. Annual excess dose was in this case $\Delta\dot{D} = 2.39 \pm 0.29$ mSv (assumed whole body exposition and majority of γ and β radiation), while calculated relative risk was $RR = 0.92 \pm 0.21$.

Cheriyan et al. 1999 [9]

The following publication describes studies conducted on newborns from the Kerala region in India. Results are given for several groups of newborns based on annual absorbed doses estimated for region where their parents live. The groups were: less than or equal to 1.50 mGy (control group), 1.51-3.00 mGy, 3.01-6.00 mGy, 6.01-12.00 mGy, 12.01-24.00 mGy and greater than 24.00 mGy. Information was provided on the average annual dose for each group, number of studied samples and cells, number of detected aberrations and relative frequency, which is basically the same as RR used here. Excess annual effective dose (again under the assumption of whole body exposure and majority of γ and β radiation), aberration incidence and relative risk have been calculated for each of the groups. For one group (12.01-24.00 mGy) RR was greater than one and because of the model's limitations (ability to model adaptive response under the assumption that it may be observed – RR being less than 1) this result had to be excluded from the data. While it was not taken into account for the model calibration, this point was included in the analysis of detrimental effects of radiation (section 4.1).

Ramachandran et al. 2013 [10]

Following paper describes studies analogous to the ones presented in Cheriyan et al. 1999. Again, persons subject to study have been divided in several annual dose groups: less than or equal to 1.50 mGy (control group), 1.51-3.00 mGy, 3.01-6.00 mGy and above 6.00 mGy. Again, for the sake of the model, whole body irradiation and majority of γ and β radiation was assumed to change

mGy to mSv. One problematic aspect here was the lack of average doses for each group other than the control group (average annual dose was 1.19 mSv). Publication, in the section regarding dosimetry refers to Jaikrishan et al.1999, who also describes studies conducted in the Kerala region, but in relation to newborn malformations. Again, they were divided into groups based on annual absorbed dose: less than or equal to 1.50 mGy (average 1.20 mGy), 1.51-3.00 mGy (average 1.81 mGy), 3.01-6.00 mGy (average 4.08 mGy), 6.01-18.00 (average 13.47 mGy) and above 18.00 mGy (average 29.53 mGy). Because of lack of dosimetric data in Ramachandran et al. 2013 and the fact, that the same dose intervals were presented in Jaikrishan et al. 1999, for the 1.51-3.00 mGy and 3.01-6.00 mGy average doses were taken from the publication Jaikrishan et al. 1999. Only for the highest interval (greater than 6.00 mGy), the dose was calculated as weighted mean of average doses from intervals 6.01-18.00 mGy and above 18.00 mGy. This resulted in annual absorbed dose of 17.99 mGy. Based on the information provided in this publication RR was calculated again for each dose interval. Dose uncertainties were assumed to be 25% of the dose value, while relative risk uncertainty was calculated based on the law of uncertainty propagation.

Mortazavi et al. 2003 [11]

Publication focused on the study of adaptive response among inhabitants of Ramsar, Iran. Mean chromosomal aberrations per cell (MCAPC) were measured before and after irradiation of blood samples with 1.5 Gy. For the calibration of the model only the data regarding MCAPC before irradiation were taken into account. Paper gives information on each of the persons separately (again, with division into HBRA and CA groups) regarding their age, cumulated dose and MCAPC. In order to get annual dose for each person, the presented cumulated dose was divided by their age. Then doses and MCAPC were averaged over each group and annual excess dose along with relative risk were calculated.

Syaifudin et al. 2018 [12]

This recent (compared to others cited above) publication describes studies conducted among the inhabitants of HBRA located in West Sulawesi, Indonesia. Study covered 88 persons in total: 70 from the HBRA of Botteng Village (who lived there at least 10 years) and 18 from control area of Keang. Similarly to the previous publication, in this case the subjects' blood samples were studied for chromosomal aberrations before and after application of 1.5 Gy dose to the sample material. Again, data used for calibration of the model included only the data regarding cells before in vitro irradiation. Dose rates presented in this paper in nSv/h were converted into mSv/year. Average annual dose from gamma radiation in the HBRA was ~5 mSv, while in the control area it was 1.4 mSv.

Part C

Publications describing studies comparing incidence or mortality in HBRA and CA

Nair et al. 1999 [13]

Publication describes results of epidemiological studies conducted between 1990 and 1996 among residents of Karunagappally area - part of Kerala region in India. Results shown in this paper describe 12 sub-areas in relation to: number of population (divided by sex), median of annual indoor and outdoor doses (separately), cancer incidence and cancer mortality among the inhabitants of each region. Publication lacks defined control area, so the area with lowest doses was assumed as such. For the purpose of model calibration, annual doses were calculated for each region as arithmetic mean of indoor and outdoor dose medians (shown in publication). Relative risk for cancer mortality and incidence (separately) was calculated as weighted mean of incidence or mortality for each sex group. Worth noting is the fact that medians of the doses given in the publication are similar to average natural background radiation doses, while their maxima are about ten times higher.

Nair et al. 2009 [14]

Decade after the publication described above, analogous paper describing cancer incidence was issued. This time, the authors instead of dividing population based on location, divided them among 5 annual dose groups: 0-0.9 mGy, 1.0-1.9 mGy, 2.0-4.9 mGy, 5.0-9.9 mGy and greater than 10 mGy. Authors provided information for each group on: average dose with one standard deviation and relative risk with 95% confidence interval (CI). Because it was not possible to include asymmetric uncertainties, the lower and higher bound uncertainties of RR were averaged (geometric mean) and divided by 2 (95% CI corresponds to two standard deviations, while in other cases one standard deviation was used).

Wei et al. 1990 [15]

In 1990 a conference organized by WHO (World Health Organisation) and IAEA (International Atomic Energy Agency) took place in Ramsar. Along with the post-conference report (International Conference on High Levels of Natural Radiation) results of epidemiological study of cancer mortality conducted among the inhabitants of Yangjiang prefecture, China in 1970-1986. Published data include average effective doses in HBRA and CA, along with respective relative risk with 95% CI. Relative risk was given for male and female population separately. This time, they were treated as two separate datapoints. Excess annual dose was calculated in a similar way as before (dose uncertainty equal to 25% of its value, followed by uncertainty propagation law). RR uncertainty was calculated in the same way as for Nair et al. 2009.

Tao et al. 2000 [16]

In 2000, another paper regarding cancer mortality among Yangjiang residents. This time, results span across years 1979-1995. Again (as in the case of Wei et al. 1990) average effective doses for CA and HBRA are given along with RR + 95% CI. Uncertainties were calculated the same way as for Wei et al. 1990.

References

[1] - Ghiassi-Nejad M., Zakeri F., Assaei R.G., Kariminia A. (2004), Long-term immune and cytogenetic effects of high level natural radiation on Ramsar inhabitants in Iran, *Journal of Environmental Radioactivity* (74): 107-116

[2] - Chen D., Wei L. (1991) Chromosome Aberration, Cancer Mortality and Hormetic Phenomena among Inhabitants in Areas of High Background Radiation in China, Journal of Radiation Research (32), Supplement 2: 46-53

[3] - Jiang T., Hayata I., Wang C., Nakai S., Yao S., Yuan Y., Dai L., Liu Q., Chen D, Wei L., Sugahara T. (2000) Dose-effect Relationship of Dicentric and Ring Chromosomes in Lymphocytes of Individuals Living in the High Background Radiation Areas in China, *Journal of Radiation Research* (41), Supplement: 63-68

[4] - Wang Z., Boice J.D.Jr., Wei L., Beebe G.W., Zha Y., Kaplan M.M., Tao Z., Maxon III H.R., Zhang S., Schneider A.B., Tan B., Wesseler T.A., Chen D., Ershow A.G., Kleinerman R.A., Littlefield L.G., Preston D. (1990) Thyroid Nodularity and Chromosome Aberrations Among Women in Areas of High Background Radiation in China, *Journal of the National Cancer Institute* (82), No. 6

[5] - Zakeri F., Rajabpour M.R., Haeri S.A., Kanda R., Hayata I., Nakamura S., Sugahara T., Ahmadpour M.J. (2011) Chromosome aberrations in peripheral blood lymphocytes of individuals living in high background radiation areas of Ramsar, Iran, *Radiation and Environmental Biophysics* (50): 571-578

[6] - Zhang W., Wang C., Chen D., Minamihisamatsu M., Morishima H., Yuan Y, Wie L., Sugahara T., Hayata I. (2003) Imperceptible Effect of Radiation Based on Stable Type Chromosome Aberrations Accumulated in the Lymphocytes of Residents in the High Background Radiation Area in China, *Journal of Radiation Research* (44): 69-74

[7] - High Background Radiation Research Group, China (1980) Health Survey in High Background Radiation Areas in China, Science, Vol. 209, 22 august 1980

[8] - Hayata I., Wang C., Zhang W., Chen D., Minamihisamatsu M., Morishima H., Yuan Y., Wei L., Sugahara T. (2000) Chromosome Translocation in Residents of the High Background Radiation Areas in Southern China, *Journal of Radiation Research* (41) supplement: 69-74

[9] - Cheryian V.D., Kurien C.J., Das B., Ramachandran E.N., Karuppasamy C.V., Thampi M.V., George K.P., Kesavan P.C., Koya P.K.M. (1999) Genetic Monitoring of the Human Population from High-Level Natural Radiation Areas of Kerala on the Southwest Coast of India. II. Incidence of Numerical and Structural Chromosomal Aberrations in the Lymphocytes of Newborns, Radiation Research (152): 154-S158

[10] - Ramachandran E., Karuppasamy C., Cheriyan V., Soren D., Das B., Anilkumar V., Koya P.K.M., Seshadri M. (2013) Cytogenetic studies on newborns from high and normal level natural radiation areas of Kerala in southwest coast of India, *International Journal of Radiation Biology* (89): 259-267

[11] - Mortazavi S.M.J., Ghiassi-Nejad M., Ikushima T., Assaie R., Heidary A., Varzegar R., Zakeri F., Asghari K., Esmaili A. (2003a) Are the Inhabitants of High Background Radiation Areas of Ramsar More Radioresistant? Scope of the Problem and the Need for Future Studies, *Iranian Journal of Radiology* 1 (1-2)

[12] - Syaifudin M., Purnami S., Rahardjo T., Kurnia I., Rahajeng N., Darlina, Nurhayati S., Mailana W., Ramadhani D., Agesti Suvifan V., Kisnanto T., Pudjadi E. (2018), Cytogenetic and Molecular Damages in Blood Lymphocyte of Inhabitants Living in High Level Natural Radiation Area (HLNRA) of Botteng Village, Mamuju, West Sulawesi, Indonesia, *Radiation Environment and Medicine 2018 Vol.7, No.2: 65-76*

[13] - Nair M.K., Nambi K.S.V., Amma N.S., Gangadharan P., Jayalekshmi P., Jayadevan S., Cherian V., Nair Reghuram K. (1999) Population Study in the High Natural Background Radiation Area in Kerala, India, *Radiation Research* (152): 145-148

[14] - Nair R.R.K., Rajan B, Akiba S., Jayalekshmi P., Nair M.K., Gangadharan P., Koga T., Morishima H., Nakamura S., Sugahara T. (2009) Background radiation and cancer incidence in Kerala, India – Karungappally cohort study, *Health Physics* (96)

[15] - Wei L., Zha Y., Tao Z., He W., Chen D., Yuan Y. (1990) Epidemiological investigation in high background radiation areas of Yangjiang, China, International Conference on High Levels of Natural Radiation, Ramsar, 3-7 Nov. 1990

[16] - Tao Z., Zha Y., Akiba S., Sun Q., Zou J., Li J., Liu Y., Kato H., Sugahara T., Wei L. (2000) Cancer Mortality in the High Background Radiation Areas of Yangjiang, China during the Period between 1979 and 1995, *Journal of Radiation Research*, 41: SUPPL., 31-41
